# CcAbl1 is required for virulence via regulating NADPH–glutathione-mediated redox balance in *Cytospora chrysosperma*

**DOI:** 10.1007/s44154-026-00302-8

**Published:** 2026-04-24

**Authors:** Wenjun Song, Ruifeng Guo, Jia Zhou, Yonglin Wang

**Affiliations:** State Key Laboratory of Efficient Production of Forest Resources, Beijing Key Laboratory for Forest Pest Control, College of Forestry, Beijing Forestry University, Beijing, 100083 China

**Keywords:** *Cytospora chrysosperma*, Carbohydrate metabolism, NADPH-glutathione homeostasis, Redox balance, Oxidative stress tolerance, Pathogenicity

## Abstract

To counteract the reactive oxygen species (ROS) derived from host plants, pathogenic fungi employ various strategies to overcome ROS or balance redox homeostasis. Glucose metabolism is vital for fungi and provides NADPH for antioxidation, but the molecular features linking glucose metabolism and redox homeostasis in fungi remain elusive. In this study, we demonstrate that CcAbl1 is indispensable for the maintenance of glucose metabolism, redox homeostasis and pathogenicity in *Cytospora chrysosperma*. Loss of *CcAbl1* severely compromises glucose utilization, disrupts carbohydrate metabolic pathways, markedly reduces intracellular NADPH levels and virulence. Furthermore, we provided evidence how CcAbl1 is involved in glucose utilization, NADPH balance, glutathione biosynthesis, and oxidative stress tolerance. These results demonstrate that CcAbl1 links virulence to NADPH and glutathione to maintain redox homeostasis in *C. chrysosperma*.

**Supplementary Information:**

The online version contains supplementary material available at 10.1007/s44154-026-00302-8.

## Introduction

Canker diseases are among the most destructive threats to woody plants worldwide, causing extensive bark deterioration, branch girdling, and even tree mortality (Yin et al. 2015; Lin et al. 2024; Meng et al. 2024; Zeng et al. 2025). Poplar canker, primarily caused by *Cytospora chrysosperma*, represents one of the most severe diseases affecting poplar production (Xu et al. 2025; Li et al. 2025). After entering host tissues through wounds, *C. chrysosperma* grows covertly within host cells, with infection hyphae expanding both intercellularly and intracellularly to colonize the bark (Yin et al. 2015). This invasion ultimately leads to tissue maceration and necrosis. Despite *C. chrysosperma* importance, how the pathogen integrates metabolic regulation with suppression of host immunity to sustain its biotrophic-like growth within poplar cells remains largely unclear. Understanding the metabolic and redox mechanisms that enable *C. chrysosperma* to survive and proliferate within host tissues is therefore essential for elucidating the development of poplar canker disease.

During pathogen invasion, fungal colonization often coincides with the breakdown of the host’s innate immune defenses (Zhang et al. 2024a, b; Zhao et al. 2024; Sun et al. 2024). A central component of this defense is the rapid accumulation of reactive oxygen species (ROS), which are generated both as by-products of cellular metabolism and as part of the host oxidative burst triggered by infection (Castro et al. 2021; Zhang et al. 2023). This ROS burst exerts potent cytotoxic effects at infection sites by oxidatively damaging DNA, proteins, and lipid membranes and by restricting the availability of nutrients required for pathogen proliferation (Sies and Jones 2020; Sachdev et al. 2021; Zhang et al. 2024a, b). In addition to their antimicrobial activity, ROS function as secondary messengers, driving the transcriptional activation of defence-related genes (Ding et al. 2024). During plant-pathogen interactions, various ROS are generated, where hydrogen peroxide (H₂O₂) playing a central role. To counteract host-derived oxidative stress, pathogens detoxify ROS via antioxidant enzymes such as catalases, peroxidases, glutathione peroxidases, and glutathione S-transferases, as well as by reducing agents including NADPH and glutathione (GSH) (Guo et al. 2019; Bomble and Nath 2022). Glutathione, the most abundant intracellular thiol, functions as a key redox buffer, donating electrons to neutralize ROS and form oxidized glutathione (GSSG). GSSG is subsequently recycled to GSH by glutathione reductase using NADPH as the electron donor (Yang et al. 2016; Feng et al. 2021). Numerous studies have highlighted the importance of these pathogen-derived antioxidant components in enabling pathogens to tolerate oxidative stress and maintain virulence (Andor et al. 2023; Cui et al. 2025; Wen et al. 2025).

Glucose serves as the pathogen’s primary carbon and energy source, supporting diverse cellular activities (Yamada and Mine 2024). Beyond its role in energy production, sugar metabolism is tightly integrated with the metabolism of proteins, nucleic acids, lipids, and secondary metabolites, underscoring its central role in coordinating and regulating overall cellular metabolism (Lastdrager et al. 2014). Furthermore, carbohydrate metabolism plays a critical role in providing the reducing power necessary to support antioxidant defense systems, thereby maintaining cellular redox balance (Fernandes and Coimbra 2023; Han et al. 2023). The pentose phosphate pathway (PPP) is the major source of NADPH, fuelling reductive systems such as glutathione, glutathione reductase, and thioredoxin (TeSlaa et al. 2023; Qiao et al. 2025). Trehalose 6-phosphate synthase 1 (*Tps1*) acts as a key metabolic sensor, detecting glucose-6-phosphate (G6P) levels and modulating NADPH production by regulating PPP activity (Wilson et al. 2010; Fernandez et al. 2012; Liu et al. 2025). In fungi such as *Saccharomyces cerevisiae* and *Magnaporthe oryzae*, disruption of *Tps1* results in severe metabolic imbalances, impaired glucose utilization and defects in development related to infection (Wilson et al. 2007; Miao et al. 2017; Van Leemputte et al. 2020). Recent work has identified *ABL1* as a downstream component of the Tps1-mediated glucose signaling pathway. *ABL1* encodes an AMP-activated protein kinase (AMPK) β-subunit-like protein, the expression of which depends on *Tps1*-mediated G6P sensing (Marroquin-Guzman et al. 2017). However, its functional contribution to carbohydrate metabolism, redox balance and pathogenicity, particularly in *C. chrysosperma*, remains largely unexplored.

In this study, we demonstrated that deletion of *CcAbl1* significantly impairs the pathogenicity of *C. chrysosperma*. Glycometabolomic analysis revealed that the loss of *CcAbl1* severely disrupted glucose metabolism, leading to a pronounced reduction in NADPH levels and perturbations in amino acid synthesis and overall metabolic activity. This disruption to glucose metabolism was associated with a significant decline in cellular glutathione content, resulting in diminished tolerance to oxidative stress. The compromised antioxidant capacity, in turn, directly restricted the fungus’s ability to colonize host tissues. Collectively, these results indicate that CcAbl1 serves as a key regulator linking glucose metabolism to redox homeostasis, thereby enabling *C. chrysosperma* to mitigate host-derived oxidative stress and successfully establish infection.

## Results

### *CcAbl1* is glucose-induced and essential for virulence

To investigate potential glucose signaling components in *C. chrysosperma*, BLASTP analysis was used to identify a single candidate gene (GME2323_g) through comparison with the protein sequence of a newly characterized glucose-inducible regulator from *Magnaporthe oryzae* (MGG_00987). Phylogenetic analysis demonstrated that *Abl1* homologues are highly conserved among diverse filamentous fungi (Fig. S1A, B). Under axenic shake conditions, *CcAbl1* expression in the wild-type (WT) strain was significantly increased by approximately ninefold-under glucose-supplemented conditions compared with glucose-free controls. These findings demonstrate that *CcAbl1* expression is regulated by glucose sensing mechanisms (Fig. S1C).

**Fig. 1:**
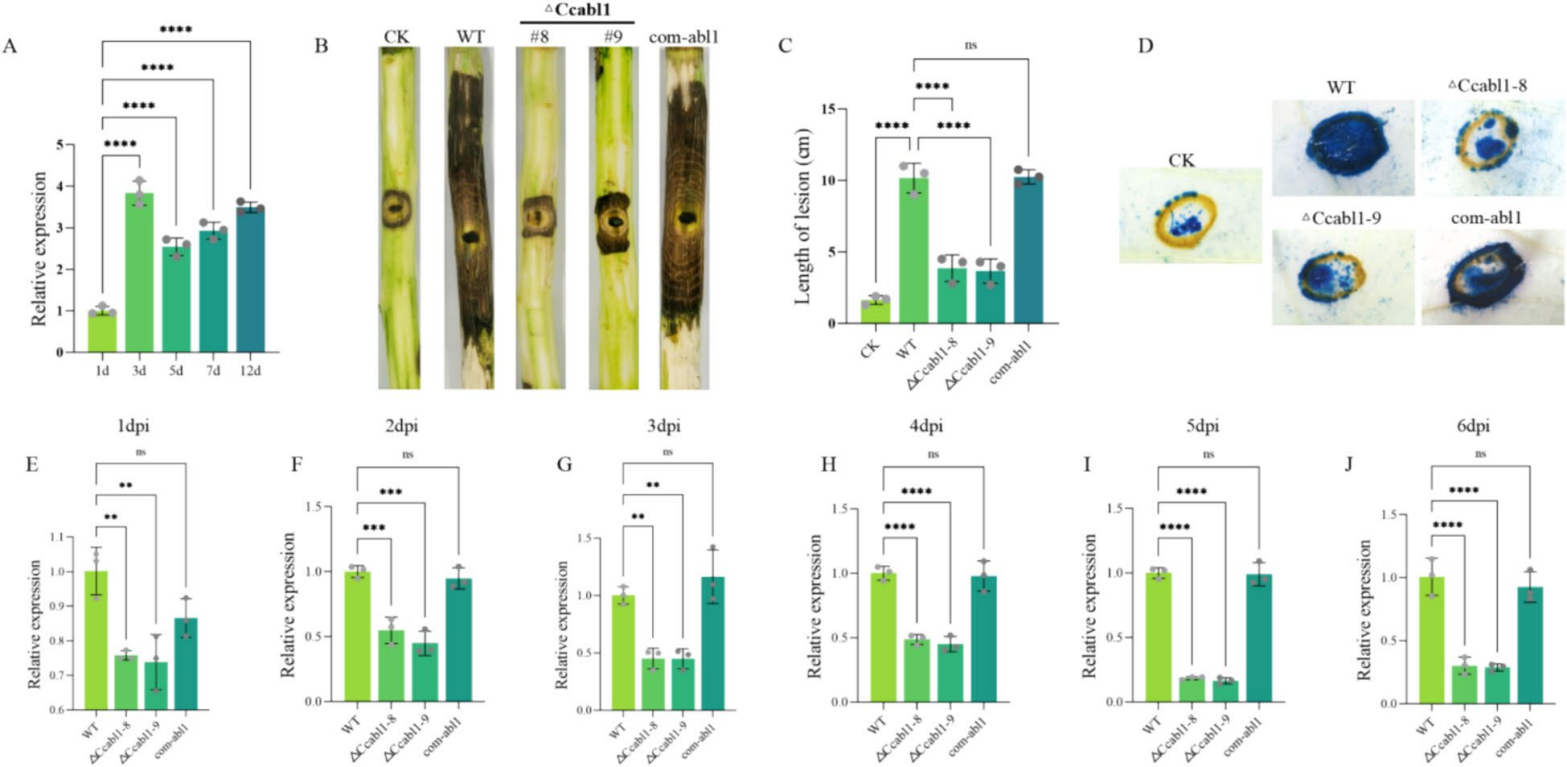
Deletion of *CcAbl1* reduces virulence. **A** Quantification of *CcAbl1* during *C. chrysosperma* infection in poplar. Data are presented as means ± SD from three technical replicates. **B** Pathogenicity assay of the WT, Δ*Ccabl1*, and com-*abl1* on poplar branches. **C** Measurement of lesion lengths on poplar branches at 7 days post-inoculation (dpi). **D** Trypan blue staining of poplar leaves infected by WT, Δ*Ccabl1*, and com-*abl1* at 24 h post-inoculation (hpi). **E-J** Quantification of fungal biomass in poplar leaves infected with WT, ∆*Ccabl1*, and com-*abl1* strains from 1 to 6 dpi

To determine the role of *CcAbl1* in the pathogenicity of *C. chrysosperma*, its transcript abundance was quantified in poplar leaves at different time points post-inoculation. Transcript levels of *CcAbl1* were low at 1 day post-infection (dpi), but were significantly upregulated at 3 dpi (Fig. 1A). This temporal expression pattern suggests that *CcAbl1* may be actively involved in processes required for early proliferation within the host. Therefore, to directly elucidate its functional role, we generated an *CcAbl1* deletion mutant (Δ*Ccabl1*) and a complementation strain (com-*abl1*) in *C. chrysosperma* through homologous recombination (Fig. S2). Pathogenicity assays performed on poplar leaves revealed that Δ*Ccabl1* produced markedly smaller necrotic lesions than the WT and the genetically complemented strain (com-*abl1*) at 7 dpi (Fig. 1B-C). Consistent with the lesion size reduction, trypan blue staining of infected leaves demonstrated a greatly diminished capacity of Δ*Ccabl1* to induce host cell death relative to WT and com-*abl1* strains (Fig. 1D). Quantitative PCR (qPCR) measurements of fungal biomass in inoculated leaves from 1 to 6 dpi further indicated a pronounced growth defect in Δ*Ccabl1*, with biomass accumulation consistently and significantly lower than in the WT and com-*abl1* strains at all tested time points (Fig. 1E-J). Collectively, these results show that CcAbl1 plays an essential role in virulence.

### *CcAbl1* is required for glucose utilization and maintenance of NADPH homeostasis

**Fig. 2:**
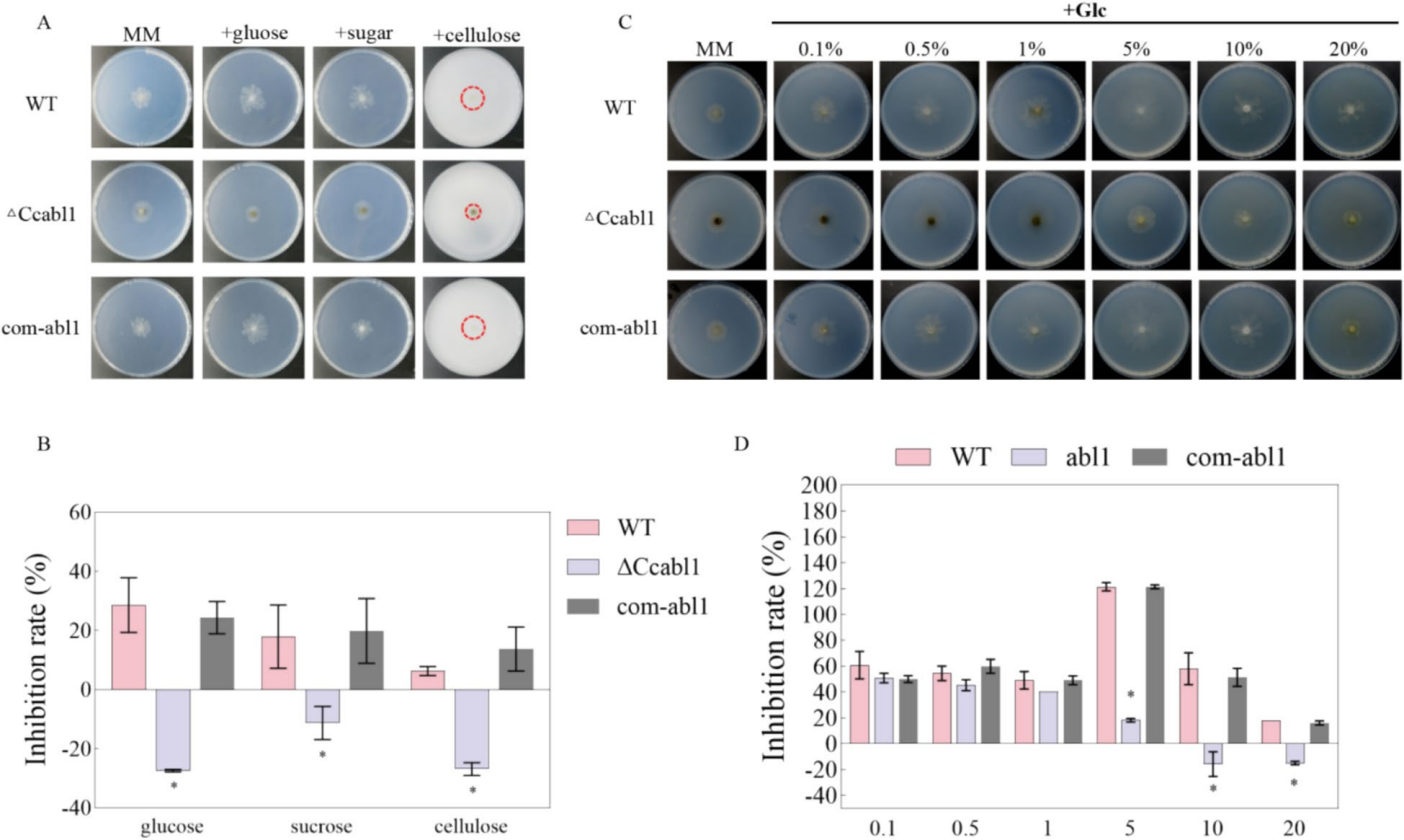
CcAbl1 is required for optimal vegetative growth on preferred carbon sources and under varying glucose availability. **A** Colony morphology of WT, ∆*Ccabl1*, and com-*abl1* strains grown on minimal medium (MM) containing 10% (w/v) glucose, sucrose, or cellulose as the sole carbon source, photographed at 10 dpi. **B** Statistical analysis of inhibition or recovery rate for WT, ∆*Ccabl1*, and com-*abl1* strains grown in MM with glucose, sucrose, or cellulose as carbon sources, respectively. **C** Colony morphology of WT, ∆*Ccabl1*, and com-*abl1* strains grown on MM containing different glucose concentrations, photographed at 10 dpi. **D** Statistical analysis of inhibition or recovery rate for WT, ∆*Ccabl1*, and com-*abl1* strains grown in MM with different glucose concentrations. Error bars represent the standard deviation, and asterisks indicate significant differences (*P* < 0.01, Student’s t-test)

In view of the glucose-responsive nature of *CcAbl1*, an investigation was conducted into its role in carbon metabolism. On minimal medium (MM) containing 10% glucose, the WT and com-*abl1* exhibited robust growth, whereas the Δ*Ccabl1* strain displayed pronounced growth defects, contrasting with the relatively weaker growth enhancement observed on non-preferred carbon sources, sucrose and cellulose (Fig. 2A-B). To further explore glucose responsiveness, cultures were inoculated onto MM containing glucose as the sole carbon source at concentrations ranging from 0 to 20%. For the WT strain and com-*abl1*, colony growth was observed to peak at 5% glucose. In contrast, the Δ*Ccabl1* mutant displayed a clear growth defect at glucose concentrations of 5% and higher (Fig. 2C-D). A series of experiments were conducted in parallel under the same glucose gradients, which revealed significant differences in culture pigmentation between WT and Δ*Ccabl1* strain (Fig. S3A-B). This suggests that there may be alterations in glucose-derived secondary metabolite production. We further quantified the total antioxidant activity in both intracellular and extracellular fractions of the WT and Δ*Ccabl1* strains following shake cultivation in minimal liquid medium containing 0%, 1%, or 5% glucose. Increasing glucose concentrations significantly enhanced total antioxidant activity in the WT strain (Fig. S4A-B). In contrast, although antioxidant activity in the Δ*Ccabl1* strain also increased with glucose availability, the magnitude of this increase was markedly lower than that observed in the WT strain (Fig. S4C-D). These results indicate that deletion of *CcAbl1* compromises glucose-dependent enhancement of antioxidant capacity, suggesting an essential role of CcAbl1 in regulating antioxidant responses under varying carbon availability.

**Fig. 3:**
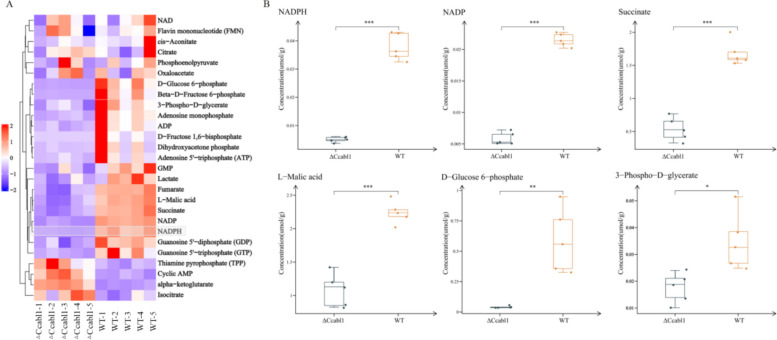
∆*Ccabl1* induces distinct metabolomic changes compared with WT strain. **A** Heatmap analysis illustrates the relative abundance of differential metabolites identified between WT and ∆*Ccabl1* strains. Metabolites with significant differences were determined using a threshold of *P* < 0.01. **B** Box plots compare the concentrations of selected differential metabolites between WT and ∆*Ccabl1* strains. Statistical significance was assessed at *P* < 0.01

To assess the impact of *CcAbl1* deficiency on downstream glucose metabolism, targeted glucose metabolomics was performed using LC–MS analysis of five biological replicates per strain. Quality control metrics confirmed the high reproducibility of metabolite quantification (relative standard deviation, RSD < 30%; Fig. S5A-B). Hierarchical clustering analysis of the metabolomic dataset revealed distinctly segregated profiles between WT and Δ*Ccabl1*, with 18 of 27 detected metabolites showing statistically significant variation (Fig. 3A). The analysis revealed that four metabolites that were elevated in the Δ*Ccabl1* sample and fourteen that were markedly reduced in comparison with the WT sample (Fig. 3B and Fig. S6). The majority of decreased metabolites were closely associated with central energy metabolism and antioxidant defense pathways. Notably, NADPH was the most dramatically diminished in Δ*Ccabl1*.

### *CcAbl1*-mediated NADPH metabolism is required for glutathione biosynthesis

**Fig. 4:**
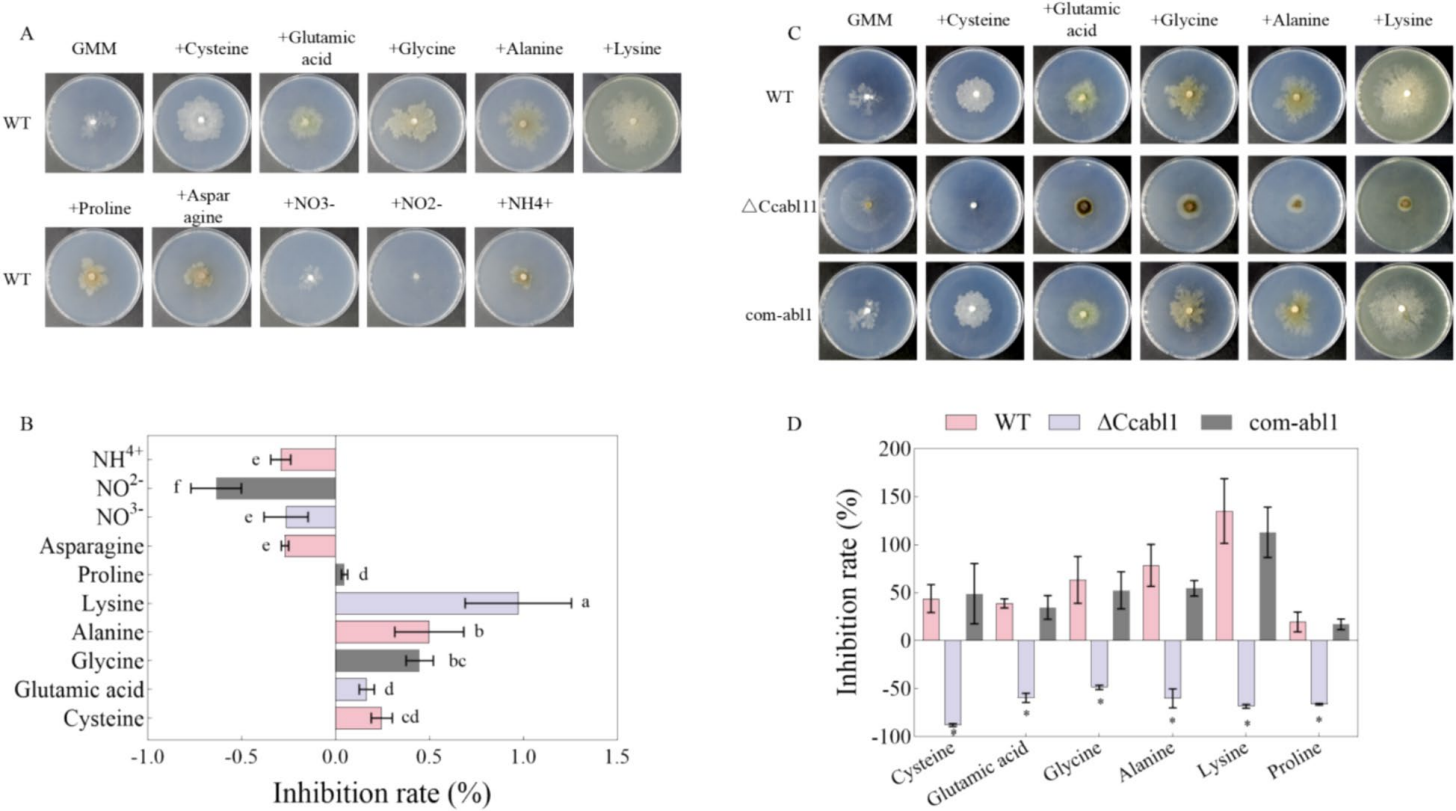
CcAbl1 is essential for the growth of *C. chrysosperma* on media with individual amino acids as the sole nitrogen source. **A** Colony morphology of WT grown on glucose minimal medium (GMM). The sole nitrogen sources were provided at 10 mM concentrations, as indicated. The strain was grown for 10 days at 25 °C. **B** Statistical analysis of inhibition or recovery rate for WT after 10 days of grown under the aforementioned conditions. Different letters indicate significant differences between these strains (*P* < 0.05, according to Duncan’s test). **C** Colony morphology of WT, Δ*Ccabl1* and com-*abl1* strains grown on GMM. The sole nitrogen sources were provided at 10 mM concentrations, as indicated. The strains were grown for 10 days at 25 °C. **D** Statistical analysis of inhibition or recovery rate for WT, Δ*Ccabl1* and com-*abl1* strains after 10 days of growth under the aforementioned conditions

As NADPH serves as a pivotal reducing equivalent for amino acid biosynthesis, nitrogen assimilation, and downstream metabolic pathways (Kim et al. 2025; Zhu et al. 2021), we investigated whether *CcAbl1* affects amino acid utilization during hyphal growth. WT hyphal growth on minimal medium supplemented individually with selected amino acids (cysteine, glutamic acid, glycine, alanine, and lysine) was significantly enhanced compared with unsupplemented controls (Fig. 4A-B). In contrast, the Δ*Ccabl1* mutant exhibited markedly impaired growth under the same conditions (Fig. 4C-D), indicating a defect in amino acid assimilation or metabolism. Notably, cysteine, glutamic acid, and glycine serve as direct substrates for glutathione biosynthesis. The inability of Δ*Ccabl1* to efficiently metabolize these amino acids, combined with its significantly reduced NADPH levels identified via glucose metabolomics (Fig. 3B), strongly implicates an impaired glutathione biosynthetic capacity.

**Fig. 5:**
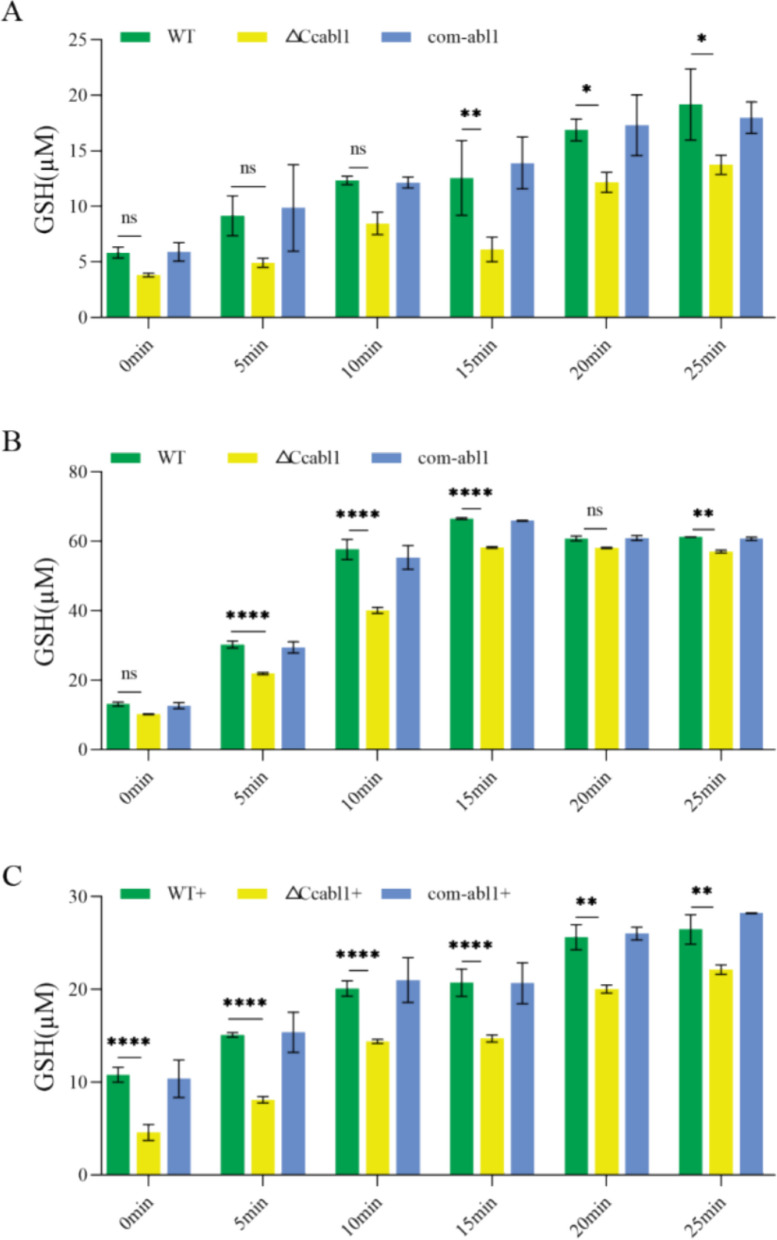
*CcAbl1* deletion changes total intracellular GSH levels across diverse growth and stress conditions. **A** Total intracellular GSH levels quantified in the indicated strains after cultivation in PDB. **B** Total intracellular GSH levels quantified in the indicated strains after cultivation in pure culture medium supplemented with 10% poplar bark. **C** Total intracellular GSH levels quantified in the indicated strains after cultivation in the presence of oxidative stress induced by H_2_O_2_. Bars represent the mean ± SD of three independent experiments. Significance was calculated relative to WT using one-way ANOVA with Duncan’s test for multiple comparisons

Quantification of total glutathione confirmed this hypothesis; the glutathione content of the Δ*Ccabl1* strain was significantly lower than in WT and com-*abl1* under both PDB and infection-mimicking conditions (10% poplar branch extract) (Fig. 5A-B). The difference became more pronounced under oxidative stress induced by hydrogen peroxide, where Δ*Ccabl1* retained substantially less total glutathione than WT and com-*abl1* (Fig. 5C). These results indicate that the loss of *CcAbl1* compromises the ability to maintain intracellular redox homeostasis, particularly in response to oxidative stress.

**Fig. 6:**
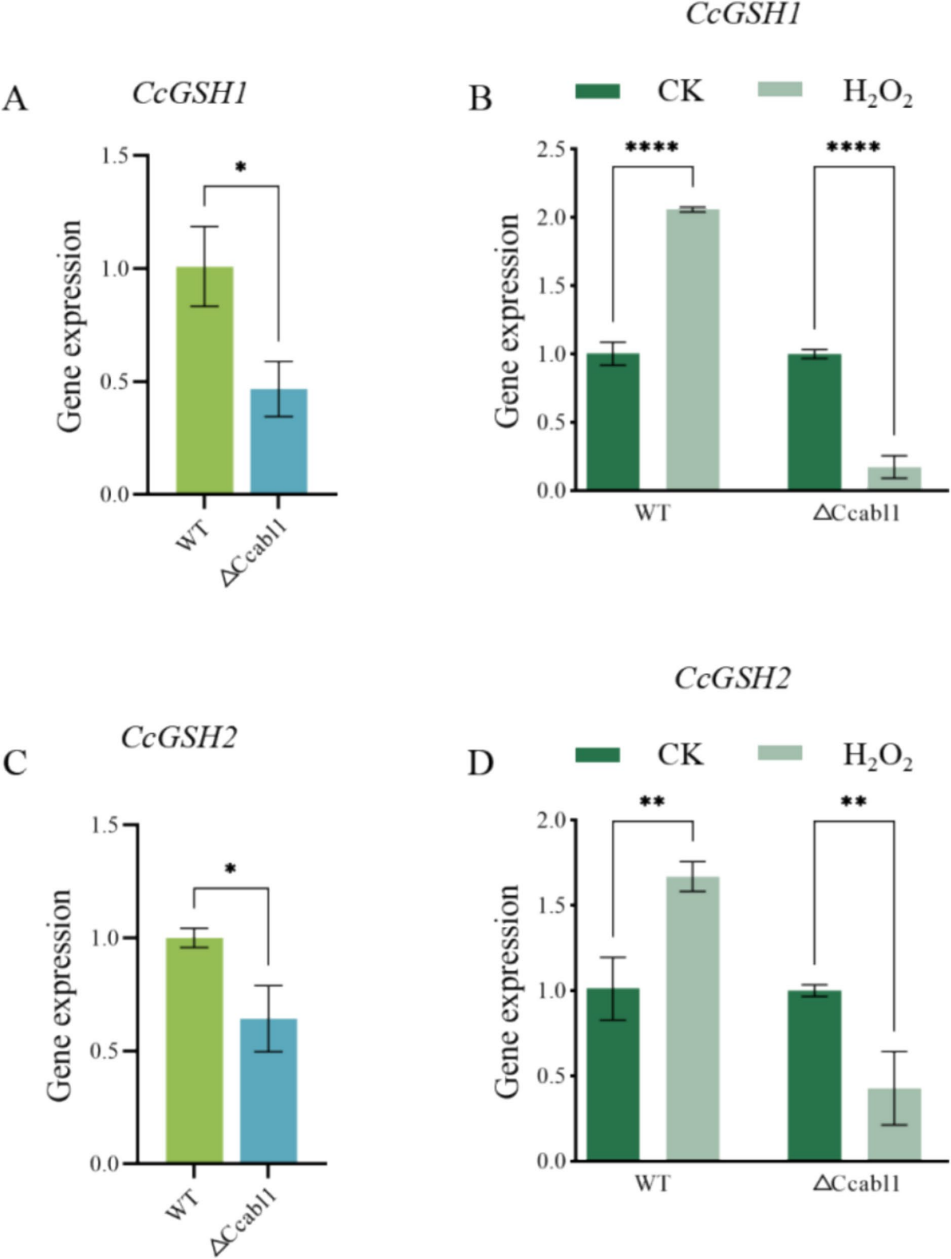
*CcAbl1* deletion changes the expression of *CcGsh1* and *CcGsh2*. **A** Expression analysis of the *CcGsh1* in WT and Δ*Ccabl1* strains was conducted after axenic growth for 5 days in medium supplemented with 10% poplar branches. B WT and Δ*Ccabl1* strains were cultivated under sterile conditions in PDB medium with or without 7.5 mM H_2_O_2_ for 10 h, after which *CcGsh1* expression levels were determined before and after hydrogen peroxide treatment. **C** Expression analysis of the *CcGsh2* in WT and Δ*Ccabl1* strains was conducted after axenic growth for 5 days in medium supplemented with 10% poplar branches. **D** WT and Δ*Ccabl1* strains were cultivated under sterile conditions in PDB medium with or without 7.5 mM H_2_O_2_ for 10 h, after which *CcGsh2* expression levels were determined before and after hydrogen peroxide treatment

To further investigate the molecular basis of the glutathione deficiency, we characterized two glutathione biosynthetic genes, *CcGsh1* and *CcGsh2*, which encode γ-glutamylcysteine synthetase and glutathione synthetase, respectively (Fig. S7). Under infection-mimicking conditions, the transcript abundances of *CcGsh1* and *CcGsh2* were significantly lower in Δ*Ccabl1* than in WT (Fig. 6A,C). Moreover, in WT, hydrogen peroxide treatment triggered a marked transcriptional induction of both genes, whereas this oxidative stress—responsive upregulation was substantially attenuated in Δ*Ccabl1* (Fig. 6B,D). Taken together, these data demonstrate that *CcAbl1* is required for NADPH-dependent glutathione pools to sustaining cellular redox balance.

### CcAbl1 is required for protection against oxidative stress

**Fig. 7:**
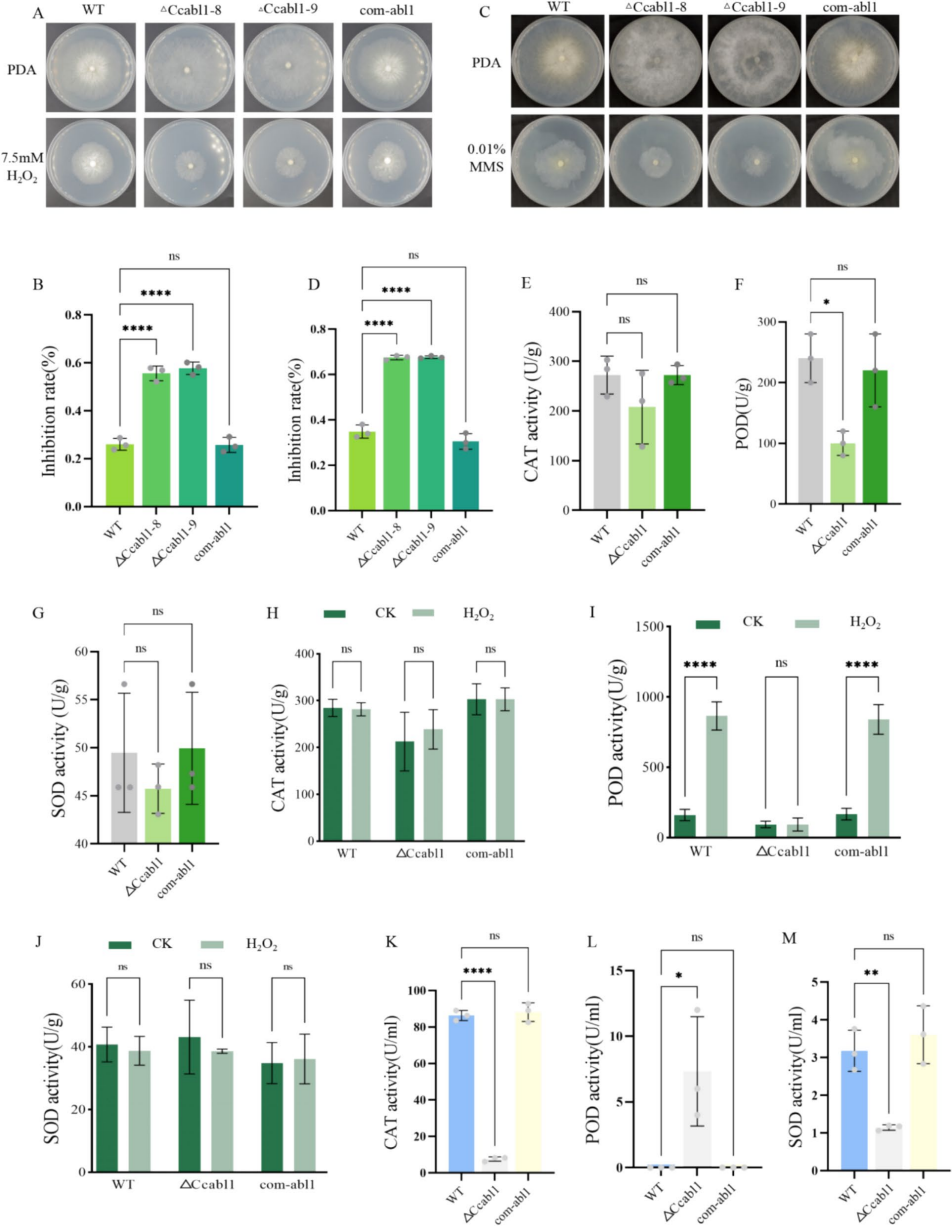
CcAbl1 is required for tolerance oxidative stress. **A** Growth phenotype of WT, Δ*Ccabl1* and com-*abl1* strains on PDA supplemented with or without H_2_O_2_ for 3 days at 25 °C. **B** Statistical analysis of the growth inhibition rates of all strains under H_2_O_2_-induced oxidative stress. **C** Growth phenotype of WT, Δ*Ccabl1* and com-*abl1* strains on PDA supplemented with or without methyl MMS for 3 days at 25 °C. **D** Statistical analysis of the growth inhibition rates of all strains under MMS-induced stress. **E–G** Quantitative measurements of catalase (CAT), peroxidase (POD), and superoxide dismutase (SOD) activities in WT, Δ*Ccabl1* and com-abl1 strains under untreated conditions. **H-J** Quantitative measurements of CAT, POD and SOD activities in WT, Δ*Ccabl1* and com-*abl1* strains following exposure to H_2_O_2_. **K-M** Quantitative measurements of CAT, POD and SOD activities in fermentation broths of WT, Δ*Ccabl1* and com-*abl1* strains after treatment with H_2_O_2_. Bars represent the mean ± standard deviation (SD) of three independent experiments. Statistical significance was calculated relative to WT using one-way ANOVA with Duncan’s correction for multiple comparisons (*P* < 0.05)

Glutathione has been identified as playing a central role in fungal oxidative stress responses (Black et al. 2024). Consistent with the reduced glutathione content observed, the Δ*Ccabl1* strain exhibited heightened sensitivity to hydrogen peroxide (Fig. 7A-B) and methyl methanesulfonate (Fig. 7C-D), indicating a comprehensive impairment in ROS tolerance. Notably, exogenous reduced GSH supplementation partially alleviated the growth defects of Δ*Ccabl1* under both H₂O₂- and methanesulfonate (MMS)-induced stress conditions (Fig. S8).

The activities of antioxidant-related enzymes (catalase, CAT; peroxidase, POD; and superoxide dismutase, SOD) were quantified in the cytoplasm of WT, Δ*Ccabl1*, and com-*abl1* strains under standard PDB growth conditions. The activities of CAT and SOD were found to be comparable among the various strains, whereas POD activity was significantly reduced in the Δ*Ccabl1* mutant relative to the WT. Subsequent to hydrogen peroxide exposure, no significant differences in intracellular CAT, POD, or SOD activities were detected (Fig. 7E-J). In contrast, extracellular enzyme assays revealed that Δ*Ccabl1* strains exhibited markedly decreased CAT and SOD activities under oxidative challenge (Fig. 7K-M). The results suggest that CcAbl1 exerts its influence over antioxidant capacity principally via extracellular ROS-scavenging enzyme systems, whereas the removal of intracellular ROS is predominantly attributable to the glutathione-dependent pathway.

### CcAbl1 is required to maintain redox balance in *C. chrysosperma* infected poplar cells to avoid triggering host innate immunity

**Fig. 8:**
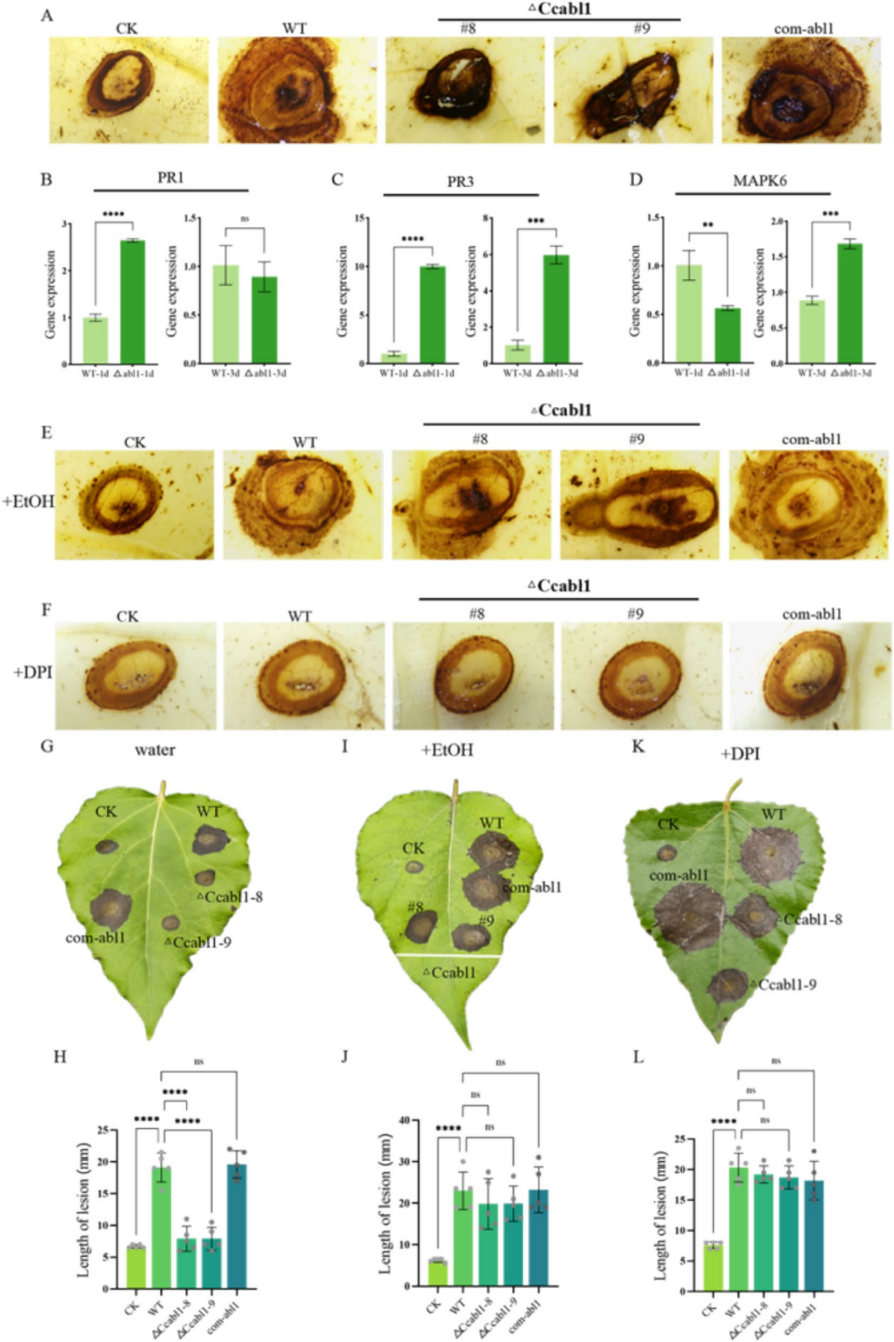
CcAbl1 is required to suppress poplar cell defenses. **A** Detection of reactive oxygen species (ROS) production in host plant leaves at 48 h post-inoculation (hpi) using DAB staining. ROS levels were quantified following infection with WT, Δ*Ccabl1*, and com-*abl1* strains. **B-D** Expression patterns of poplar defense-related genes (*PR-1*, *PR-3*, and *MAPK6* genes) during infection with WT and Δ*Ccabl1* strains, measured at the indicated time points. **E–F** Detection of ROS production in host plant leaves at 48 hpi using DAB staining. ROS levels were quantified following infection with WT, Δ*Ccabl1*, and com-*abl1* strains under treatments with ethanol (**E**), or diphenyleneiodonium chloride (DPI) (**F**). **G-L** Lesion lengths measured in poplar leaves infected with WT, Δ*Ccabl1*, and com-*abl1* strains under treatments with H_2_O (control) (**G**,**H**), ethanol (**I**,**J**), or DPI (**K**,**L**) at 4 dpi. Error bars represent the mean ± standard error (SE) of three independent experiments. Statistical significance was determined using one-way ANOVA, with lowercase letters indicating significant differences (*P* < 0.05) between treatments, and asterisks denoting significance levels (***, *P* < 0.001; **, *P* < 0.01; *, *P* < 0.05)

To further investigate poplar defense responses upon challenge with the Δ*Ccabl1* mutant, typical plant defense pathways were assessed. During the infection process, the Δ*Ccabl1* mutant was found to elicit significantly more pronounced ROS bursts in the host when compared to the WT and com-*abl1* strain (Fig. 8A). Transcript levels of the poplar pathogenesis-related (PR) genes PR1 and PR3, together with MAPK6 (a key regulator of host defense signalling), were consistently and significantly upregulated in plants infected with the mutant (Fig. 8B-D). This finding indicates that the mutant's impaired oxidative stress tolerance may contribute to heightened host immune activation.

To assess the contribution of host-derived ROS to reduced mutant virulence, we suppressed host oxidative responses. First, pretreatment of host tissues with ethanol, which rapidly kills host cells and disrupts ROS generation, substantially restored pathogenicity of the mutant to near WT levels (Fig. 8E and G-J). Second, pharmacological inhibition of host NADPH oxidase (NOX) activity using diphenyleneiodonium (DPI) significantly reduced hydrogen peroxide accumulation in infected tissues and partially rescued mutant virulence (Fig. 8F and K-L). Collectively, these observations directly associate the reduced virulence of Δ*Ccabl1* with its heightened sensitivity to host-generated oxidative stress, indicating that *CcAbl1* confers an essential capacity to withstand plant ROS-mediated defense during infection.

## Discussion

This study establishes *CcAbl1* as a central glucose-sensing regulator that coordinates carbon metabolism, redox homeostasis, and pathogenicity in *C. chrysosperma*. The disruption of *CcAbl1* resulted in a marked attenuation of virulence, accompanied by substantial perturbation of glucose metabolism and depletion of intracellular NADPH. This reduction in NADPH was directly associated with a significant decrease in glutathione (GSH) levels, thereby weakening the pathogen’s capacity to detoxify reactive oxygen species (ROS) and impeding its capacity to effectively colonise host tissue or expand necrotic lesions. These findings underscore a tight mechanistic coupling between glucose sensing, NADPH-dependent antioxidant defence, and fungal pathogenicity.

Sugars function not only as primary carbon and energy sources but also act as critical modulators of stress responses (Li et al. 2024). Under oxidative stress, metabolic flux often shifts from glycolysis towards the pentose phosphate pathway (PPP), enabling increased NADPH production to sustain antioxidant capacity (Aburto et al. 2025). NADPH provides reducing equivalents required for the regeneration of antioxidant systems, particularly through GSH cycling, thereby facilitating the neutralisation of ROS (Ryder et al. 2013). Analogous to the ABL1 gene in the*Magnaporthe oryzae*, which encodes a protein functionally related to the AMPK β subunit, CcAbl1 is glucose-inducible and plays a critical role in glucose metabolism. In this study, deletion of *CcAbl1* impaired this metabolic reprogramming. Specifically, Δ*Ccabl1* mutants exhibited a significant reduction in G-6-P, the entry metabolite of the PPP, accompanied by a marked decrease in NADPH levels (Fig. 3). These results indicate that CcAbl1 contributes to NADPH homeostasis, likely by modulating PPP flux. Metabolomic analyses revealed a significant depletion of organic acids and intermediates, including fumarate, α‑ketoglutarate, guanosine monophosphate, isocitrate, lactate, malate, and succinate. Many of these have been linked to antioxidant and redox processes. The data presented herein suggest that *CcAbl1* exerts a regulatory influence not only over flux through glycolysis and the PPP, but also over organic acid metabolism. This latter facet, being of considerable interest, warrants further investigation in order to ascertain its potential role in pH regulation and virulence.

Glutathione is a tripeptide that functions serving as a primary non‑enzymatic antioxidant. It has the capacity to directly scavenge ROS and act as a cofactor for multiple antioxidant enzymes, including glutathione reductase, glutathione peroxidase, and glutathione S‑transferases (Cassier-Chauvat et al. 2023; Ju et al. 2025). Its biosynthesis and recycling are dependent on substrate amino acids and NADPH-derived reducing power (Dorion et al. 2021; Hristov 2022). The results of the study demonstrated that the deletion of the *CcAbl1* gene in *C. Chrysosperma* led to a significant reduction in the fungus's ability to utilise amino acids that serve as precursors for glutathione biosynthesis, particularly glutamic acid, cysteine and glycine (Fig. 4). Consistent with this metabolic defect, the Δ*Ccabl1* led to a significant decrease in intracellular glutathione levels, an effect that was further compounded under conditions of oxidative stress (Fig. 5). The depletion of glutathione resulted in a critical impairment of the strain's antioxidant defences, rendering it hypersensitive to hydrogen peroxide and DNA‑damaging agents, and sharply diminishing its ability to detoxify reactive oxygen species. Enzyme activity assays revealed that catalase (CAT) and superoxide dismutase (SOD) activities remained unaltered by *CcAbl1* loss under both normal and H₂O₂‑induced conditions (Fig. 7E-J). In contrast, POD activity was significantly reduced in the mutant, implicating *CcAbl1* in the specific regulation of glutathione‑dependent antioxidant systems and POD‑mediated ROS detoxification. Collectively, these findings position *CcAbl1* as a key regulator of redox homeostasis in *C. Chrysosperma*, acting predominantly through the modulation of glutathione metabolism.

During the process of plant infection, the host tissues typically generate a rapid oxidative burst, releasing high levels of ROS to impede pathogen colonisation (Zhang et al. 2024a, b). Excessive ROS accumulation within fungal cells has been demonstrated to damage nucleic acids, proteins, and membranes, ultimately causing cell death (Kang et al. 2021). To survive this oxidative onslaught, successful pathogens deploy multiple strategies, including the activation of antioxidant enzyme systems, the suppression of host ROS production, and enhancement of intrinsic oxidative stress tolerance (Zhang et al. 2023; Yuan et al. 2021). Such countermeasures are critical for neutralising host-derived ROS and enabling sustained infection. In this experiment, the deletion of *CcAbl1* led to a significant decrease in both the extracellular CAT and SOD activities under conditions of oxidative stress, as well as an increase in ROS accumulation within the host tissue (Fig. 7K, M). This effect was revealed through the use of DAB staining (Fig. 8A). Such conditions triggered stronger expression of host defence‑related genes (Fig. 8B-D) and caused more severe cellular damage in fungal tissue. It is notable that treatment with the NADPH oxidase inhibitor DPI partially restored the virulence of the ∆*Ccabl1* strain (Fig. 8G-I), confirming that its attenuated pathogenicity is primarily attributable to heightened sensitivity to hydrogen peroxide arising from impaired NADPH‑GSH antioxidant capacity. This loss of resilience under oxidative conditions restricts lesion expansion and limits the pathogen’s capacity to establish and maintain in planta infections.

In summary, the present study demonstrates that *CcAbl1* functions as a critical upstream regulator in *C. chrysosperma*, playing an essential role in coordinating carbohydrate metabolism, driving NADPH production, and modulating the glutathione-dependent antioxidant defense system to sustain oxidative homeostasis. The absence of *CcAbl1* has been demonstrated to disrupt metabolic flux within the pentose phosphate pathway, resulting in NADPH depletion and diminished antioxidant reserves. This, in turn, has been shown to significantly impair the pathogen's ability to tolerate ROS and survive under oxidative stress. Additionally, *CcAbl1* deficiency exacerbates host immune responses, thereby further constraining fungal colonization. These findings firmly establish *CcAbl1* as a central regulatory node that integrates metabolic control with pathogenic success, underscoring the pivotal role of the glucose-responsive NADPH-GSH redox system in shaping plant-pathogen interactions.

## Conclusion

In conclusion, we identify CcAbl1 as a glucose-responsive regulator that links central metabolism to virulence in *Cytospora chrysosperma* through redox homeostasis maintenance. This work reveals that necrotrophic fungi harness glucose metabolism to fuel ROS detoxification systems essential for overcoming plant immunity. Beyond mechanistic insights, our findings suggest that targeting the metabolic-redox interface may offer novel strategies for controlling poplar *Cytospora* canker and related fungal diseases affecting economically important crops and forests.

## Materials and methods

### Fungal strains and culture conditions

The wild-type (WT) strain of *Cytospora chrysosperma* (CFCC 89981), which was isolated from Populus and obtained from the China Forestry Culture Collection Center, was used in this study. Fungal strains were maintained on potato dextrose agar (PDA) at 25 °C or grown in potato dextrose broth (PDB) at 25 °C with shaking at 150 rpm.

### Deletion and complementation of *CcAbl1* in *C. chrysosperma*

Targeted deletion mutants of *CcAbl1* were generated using the split-marker approach and transformed via the previously described PEG-mediated method (Xie et al. 2023). The entire open reading frame (ORF) of *CcAbl1* was replaced with a hygromycin resistance gene. The upstream and downstream flanking sequences were amplified using specific primers (Table S1) and fused to the hygromycin cassette via fusion PCR. To complement the deletion, the full coding region of *CcAbl1*, including its native promoter and terminator, was amplified and introduced into the deletion strains (Table S1).

### Virulence assays and analyses of infection

Virulence was assessed on one-year-old poplar branches. Briefly, 5-mm wounds were created with a hot iron rod, and 5-mm fungal agar plugs were inoculated into the wounds. Infections were maintained for 7 days (Li et al. 2025). Leaf infection assays were performed similarly to twig inoculations.

### LC–MS ion monitoring analysis

Liquid chromatography-mass spectrometry (LC–MS) was used to analyze metabolic changes in WT and Δ*Ccabl1* strains during glucose metabolism. Total ion current (TIC) chromatograms demonstrated strong signal intensity, high peak capacity, consistent retention times, and excellent reproducibility (Fig. S4A). Quality control (QC) samples, prepared by pooling equal portions of all samples, showed relative standard deviation (RSD) values below 30%, indicating data stability and reliability (Fig. S4B).

### Stress sensitivity assay

To evaluate stress responses, 5 mm mycelial plugs from the WT, Δ*Ccabl1*, and complemented (com-*abl1*) strains were transferred from PDA to modified PDA plates containing 7.5 mM H_2_O_2_ or 0.01% methyl methanesulfonate (MMS). To assess whether glutathione supplementation could alleviate oxidative damage associated with the Δ*Ccabl1* mutant, the same strains were inoculated onto stress‑containing plates additionally supplemented with 0.5 mM reduced glutathione. Plates were incubated at 25 °C in the dark for 3 days, with PDA plates lacking stress agents serving as controls. Colony diameters were measured to calculate growth and inhibition rates, with inhibition determined as (CK − growth under treatment)/CK.

### RNA extraction and RT-qPCR

Total RNA was extracted from *C. chrysosperma* samples at various developmental stages and from transformants, using the Total RNA Kit (Vazyme, Nanjing, China). RNA integrity was confirmed by agarose gel electrophoresis. First-strand cDNA was synthesized using oligo(dT) primers and M-MLV reverse transcriptase kit (Vazyme, Nanjing, China). Relative gene expression was quantified using the 2^⁻ΔΔCt^ method with at least three biological replicates.

To monitor *CcAbl1* expression during infection, RNA was extracted from poplar leaf tissue inoculated with the WT strain at 1, 3, 5, 7, and 12 days post-inoculation (dpi). *CcActin* served as an internal reference. Fungal biomass during infection by WT, Δ*Ccabl1*, and com-*abl1* strains was assessed by extracting genomic DNA (TIANGEN, Beijing, China) from inoculated leaves at 1–6 dpi, using *CcActin* as a reference. The expression of two classes of PR genes in *Populus canadensis* twig tissues was analyzed at 1 and 3 dpi, using EF1-alpha (GenBank XM_002316315) as the internal control.

### Total glutathione quantification

For total glutathione measurement, mycelial blocks were inoculated into liquid PDB and incubated at 25 °C for 5 days with shaking at 150 rpm. Fungal mycelia were collected by vacuum filtration, washed three times with sterile water, and ground thoroughly in liquid nitrogen. Approximately 0.01 g of mycelial powder was mixed with 100 µL protein removal reagent and centrifuged at 1000 × g for 10 min. Supernatants were used to quantify total glutathione (GSSG/GSH) using a commercial assay kit (Beyotime, Shanghai, China). All experiments were conducted with three biological replicates.

### Antioxidant enzyme activity assays

To assess the activities of antioxidant enzymes, mycelia from the WT, Δ*Ccabl1*, and com-*abl1* strains were inoculated into PDB and cultured for 5 days. Mycelia and culture filtrates were collected and analyzed using commercial enzyme activity kits (Suzhou Keming Biotechnology Co., Ltd., Suzhou, China).

For assays under hydrogen peroxide-induced stress, 3-day-old cultures were treated with 7.5 mM H_2_O_2_, and supernatants were collected at 0, 0.5, 1, 2, 4, and 10 h post-treatment for analysis using the same enzyme kits.

### Detection of reactive oxygen

ROS burst were visualized using the DAB staining approach as described previously (Xu et al. 2022). A Leica DM2500 microscope (Wetzlar, Germany) was used for the observations.

### ABTS assay for total antioxidant capacity

WT and Δ*Ccabl1* strains were cultivated in shake flasks containing minimal medium supplemented with glucose at final concentrations of 0%, 1%, or 5% (w/v). After incubation, mycelia and extracellular culture supernatants (fermentation broths) were harvested separately. Total antioxidant capacity was determined using a Total Antioxidant Capacity Assay Kit (Beyotime, Shanghai, China) according to the manufacturer’s instructions.

## Supplementary Information

Supplementary Material 1.

## Data Availability

The raw sequencing data have been deposited in the China National Center for Bioinformation, Beijing Institute of Genomics, Chinese Academy of Sciences (Accession No. OMIX013901) that are publicly accessible at https://ngdc.cncb.ac.cn/.

## Abbreviations

WT: Wild-type
com-abl1: Complemented abl1 strain
PDA: Potato dextrose agar
PDB: Potato dextrose broth
ROS: Reactive Oxygen Species
DAB: 3,3’-Diaminobenzidine
CAT: Catalase
POD: Peroxidase
SOD: Superoxide dismutase
PPP: Pentose phosphate pathway
GSH: Glutathione
H₂O₂: Hydrogen peroxide
GSSG: Oxidized glutathione
Tps1: Trehalose 6-phosphate synthase 1
G6P: Glucose-6-phosphate
AMPK: AMP-activated protein kinase
dpi: Day post-infection
Δ*Ccabl1*: CcAbl1 deletion mutant
qPCR: Quantitative PCR
MM: Minimal medium
PR: Poplar pathogenesis-related
DPI: Diphenyleneiodonium
NOX: NADPH oxidase
LC–MS: Liquid chromatography-mass spectrometry
TIC: Total ion current
QC: Quality control
RSD: Relative standard deviation
MMS: Methyl methanesulfonate

## References

[CR1] Aburto C, Ruminot I, San Martin A (2025) Acute stimulation of glucose metabolism by H_2_O_2_ sustains the NADPH steady-state under oxidative stress. Redox Biol 85:103740. 10.1016/j.redox.2025.103740 40609477 10.1016/j.redox.2025.103740 PMC12271791

[CR2] Andor A, Mohanraj M, Pato ZA, Uri K, Biri-Kovacs B, Cheng Q, Arner ESJ (2023) TXNL1 has dual functions as a redox active thioredoxin-like protein as well as an ATP - and redox - independent chaperone. Redox Biol 67:15. 10.1016/j.redox.2023.102897 10.1016/j.redox.2023.102897 PMC10570131 37804695

[CR3] Black B, Silva LBRD, Hu G, Qu X, Smith DFQ, Magaña AA, Horianopoulos LC, Caza M, Attarian R, Foster LJ, Casadevall A, Kronstad JW (2024) Glutathione-mediated redox regulation in *Cryptococcus neoformans* impacts virulence. Nat Microbiol 9(8):2084–2098. 10.1038/s41564-024-01721-x 38956248 10.1038/s41564-024-01721-x PMC11930340

[CR4] Bomble P, Nath BB (2022) Differential manifestation of RONS and antioxidant enzymes in response to singular versus combinatorial stress in *Chironomus ramosus*. Stress Biol 2(1):14. 10.1007/s44154-022-00077-8 37676561 10.1007/s44154-022-00077-8 PMC10442003

[CR5] Cassier-Chauvat C, Marceau F, Farci S, Ouchane S, Chauvat F (2023) The glutathione system : a journey from *Cyanobacteria* to higher eukaryotes. Antioxidants 12(6):32. 10.3390/antiox12061199 10.3390/antiox12061199 PMC10295655 37371929

[CR6] Castro B, Citterico M, Kimura S, Stevens DM, Wrzaczek M, Coaker G (2021) Stress-induced reactive oxygen species compartmentalization, perception and signalling. Nat Plants 7(4):403–412. 10.1038/s41477-021-00887-0 33846592 10.1038/s41477-021-00887-0 PMC8751180

[CR7] Cui X, Zhang D, Gao L, Liu N, Lian S, Ren W, Li B, Wang C (2025) β-Glucosidase VmGlu1 is required for toxin production and pathogenicity of *Valsa mali*. Phytopathology Res 7(1):12. 10.1016/j.nanoen.2021.105878

[CR8] Ding Y, Yan B, Zhao S, Chen Y, Wan H, Qian W (2024) Synthetic modulation of ROS scavenging during host - *Sclerotinia sclerotiorum* interaction : a new strategy for the development of highly resistant plants. Phytopathol Res 6(1):13. 10.1186/s42483-024-00238-9

[CR9] Dorion S, Ouellet JC, Rivoal J (2021) Glutathione metabolism in plants under stress: beyond reactive oxygen species detoxification. Metabolites 11(9):641. 10.3390/metabo11090641 34564457 10.3390/metabo11090641 PMC8464934

[CR10] Feng H, Xu M, Gao Y, Liang J, Guo F, Guo Y, Huang L (2021) Vm-milR37 contributes to pathogenicity by regulating glutathione peroxidase gene* VmGP *in *Valsa mali*. Mol Plant Pathol 22(2):243–254. 10.1111/mpp.13023 33278058 10.1111/mpp.13023 PMC7814965

[CR11] Fernandes PAR, Coimbra MA (2023) The antioxidant activity of polysaccharides : a structure-function relationship overview. Carbohydr Polym 314:15. 10.1016/j.carbpol.2023.120965 10.1016/j.carbpol.2023.120965 37173007

[CR12] Fernandez J, Wright JD, Hartline D, Quispe CF, Madayiputhiya N, Wilson RA (2012) Principles of carbon catabolite repression in the rice blast fungus : Tps1, Nmr1-3, and a MATE - family pump regulate glucose metabolism during infection. PLoS Genet 8(5):29. 10.1371/journal.pgen.1002673 10.1371/journal.pgen.1002673 PMC3342947 22570632

[CR13] Guo Y, Yao S, Yuan T, Wang Y, Zhang D, Tang W (2019) The spatiotemporal control of KatG2 catalase-peroxidase contributes to the invasiveness of *Fusarium graminearum* in host plants. Mol Plant Pathol 20(5):685–700. 10.1111/mpp.12785 30919582 10.1111/mpp.12785 PMC6637876

[CR14] Han Y, Xu T, Chen H, Tang M (2023) Sugar metabolism and 14-3-3 protein genes expression induced by arbuscular mycorrhizal fungi and phosphorus addition to response drought stress in *Populus cathayana*. J Plant Physiol 288:154075–154075. 10.1016/j.jplph.2023.154075 37643547 10.1016/j.jplph.2023.154075

[CR15] Hristov BD (2022) The role of glutathione metabolism in chronic illness development and its potential use as a novel therapeutic target. Cureus 14(9):e29696. 10.7759/cureus.29696 36321012 10.7759/cureus.29696 PMC9616098

[CR16] Ju Y, Zhang Y, Tian X, Zhu N, Zheng Y, Qiao Y, Yang T, Niu B, Li X, Yu L, Liu Z, Wu Y, Zhi Y, Dong Y, Xu Q, Yang X, Wang X, Wang X, Deng H, Mao Y, Li X (2025) Protein S-glutathionylation confers cellular resistance to ferroptosis induced by glutathione depletion. Redox Biol 83:103660. 10.1016/j.redox.2025.103660 40354766 10.1016/j.redox.2025.103660 PMC12139022

[CR17] Kang J, Bishayee K, Huh S (2021) Azoxystrobin impairs neuronal migration and induces ROS dependent apoptosis in cortical neurons. Int J Mol Sci 22(22):12495. 10.3390/ijms222212495 34830376 10.3390/ijms222212495 PMC8622671

[CR18] Kim D, Kesavan R, Ryu K, Dey T, Marckx A, Menezes C, Praharaj PP, Morley S, Ko B, Soflaee MH, Tom HJ, Brown H, Vu HS, Tso S, Brautigam CA, Lemoff A, Mettlen M, Mishra P, Cai F, Allen DK, Hoxhaj G (2025) Mitochondrial NADPH fuels mitochondrial fatty acid synthesis and lipoylation to power oxidative metabolism. Nat Cell Biol 27(5):790–800. 10.1038/s41556-025-01655-4 40258949 10.1038/s41556-025-01655-4 PMC12331256

[CR19] Lastdrager J, Hanson J, Smeekens S (2014) Sugar signals and the control of plant growth and development. J Exp Bot 65(3):799–807. 10.1093/jxb/ert474 24453229 10.1093/jxb/ert474

[CR20] Li M, Liu X, Wu F, Shi X, Kong D, Li X, Mu C, Qu D, Wang L, Su H (2024) Fermentation broth of a novel endophytic fungus enhanced maize salt tolerance by regulating sugar metabolism and phytohormone biosynthesis or signaling. Plant Physiol Biochem 216:109125. 10.1016/j.plaphy.2024.109125 39278049 10.1016/j.plaphy.2024.109125

[CR21] Li Q, Guo R, Li A, Wang Y (2025) Roles of NADPH oxidases in regulating redox homeostasis and pathogenesis of the poplar canker fungus *Cytospora chrysosperma*. Stress Biol 5(1):33. 10.1007/s44154-025-00223-y 40338399 10.1007/s44154-025-00223-y PMC12061831

[CR22] Lin L, Fan XL, Groenewald JZ, Jami F, Wingfield MJ, Voglmayr H, Jaklitsch W, Castlebury LA, Tian CM, Crous PW (2024) *Cytospora* : an important genus of canker pathogens. Stud Mycol 109:323–401. 10.3114/sim.2024.109.05 39717654 10.3114/sim.2024.109.05 PMC11663427

[CR23] Liu Y, Li B, Chen T, Tian S, Zhang Z (2025) The synthesis, degradation and biological function of trehalose-6-phosphate. Stress Biol 5(1):38–38. 10.1007/s44154-025-00235-8 40445466 10.1007/s44154-025-00235-8 PMC12125463

[CR24] Marroquin-Guzman M, Sun G, Wilson RA (2017) Glucose-ABL1-TOR signaling modulates cell cycle tuning to control terminal appressorial cell differentiation. PLoS Genet 13(1):e1006557. 10.1371/journal.pgen.1006557 28072818 10.1371/journal.pgen.1006557 PMC5266329

[CR25] Meng Y, Xiao Y, Zhu S, Xu L, Huang L (2024) VmSpm1: a secretory protein from *Valsa mali* that targets apple’s abscisic acid receptor MdPYL4 to suppress jasmonic acid signaling and enhance infection. New Phytol 244(6):2489–2504. 39417426 10.1111/nph.20194

[CR26] Miao Y, Tenor JL, Toffaletti DL, Maskarinec SA, Liu J, Lee RE, Perfect JR, Brennan RG (2017) Structural and in vivo studies on trehalose-6-phosphate synthase from pathogenic fungi provide insights into Its catalytic mechanism, biological necessity, and potential for novel antifungal drug design. Mbio 8(4):10–1128. 10.1128/mBio.00643-17 10.1128/mBio.00643-17 PMC5527307 28743811

[CR27] Qiao J, Yu Z, Zhou H, Wang W, Wu H, Ye J (2025) The pentose phosphate pathway : from mechanisms to implications for gastrointestinal cancers. Int J Mol Sci 26(2):26. 10.3390/ijms26020610 10.3390/ijms26020610 PMC11765532 39859324

[CR28] Ryder LS, Dagdas YF, Mentlak TA, Kershaw MJ, Thornton CR, Schuster M, Chen J, Wang Z, Talbot NJ (2013) NADPH oxidases regulate septin-mediated cytoskeletal remodeling during plant infection by the rice blast fungus. Proc Natl Acad Sci U S A 110(8):3179–3184. 10.1073/pnas.1217470110 23382235 10.1073/pnas.1217470110 PMC3581893

[CR29] Sachdev S, Ansari SA, Ansari MI, Fujita M, Hasanuzzaman M (2021) Abiotic stress and reactive oxygen species : generation , signaling , and defense mechanisms. Antioxidants 10(2):37. 10.3390/antiox10020277 10.3390/antiox10020277 PMC7916865 33670123

[CR30] Sies H, Jones DP (2020) Reactive oxygen species (ROS) as pleiotropic physiological signalling agents. Nat Rev Mol Cell Biol 21(7):363–383. 10.1038/s41580-020-0230-3 32231263 10.1038/s41580-020-0230-3

[CR31] Sun G, Xia Y, Li K, Zhu Q, Ding F, Gu H, Zhang Z, Li X, Mi X, Chen J, Yao R, Zhang S, Ouyang H, Chen X, Liu T, Jiang H, Zhao Y, Qiu M, Ye W, Duan K, Ma Z, Dong S, Yin H, Wang Y, Wang Y (2024) Dual activation of soybean resistance against *Phytophthora sojae* by pectin lyase and degraded pectin oligosaccharides. Sci China Life Sci 67(12):2746–2760. 10.1007/s11427-024-2724-5 39549112 10.1007/s11427-024-2724-5

[CR32] TeSlaa T, Ralser M, Fan J, Rabinowitz JD (2023) The pentose phosphate pathway in health and disease. Nat Metab 5(8):1275–1289. 10.1038/s42255-023-00863-2 37612403 10.1038/s42255-023-00863-2 PMC11251397

[CR33] Van Leemputte F, Vanthienen W, Wijnants S, Van Zeebroeck G, Thevelein JM (2020) Aberrant intracellular pH regulation limiting glyceraldehyde - 3-phosphate dehydrogenase activity in the glucose-sensitive yeast tps1Δ mutant. Mbio 11(5):18. 10.1128/mBio.02199-20 10.1128/mBio.02199-20 PMC7593968 33109759

[CR34] Wen X, Li H, Li J, Mapuranga J, Zhang N, Song L, Chang J, Li R, Zhang Y, Liu D, Yang W (2025) The *Puccinia triticina* effector Pt3372 suppresses wheat innate immunity by targeting wheat TaERP3 in TcLr2a and TcLr18. Phytopathol Res 7(1):15. 10.1186/s42483-025-00332-6

[CR35] Wilson RA, Jenkinson JM, Gibson RP, Littlechild JA, Wang Z, Talbot NJ (2007) Tps1 regulates the pentose phosphate pathway, nitrogen metabolism and fungal virulence. EMBO J 26(15):3673–3685. 10.1038/sj.emboj.7601795 17641690 10.1038/sj.emboj.7601795 PMC1949003

[CR36] Wilson RA, Gibson RP, Quispe CF, Littlechild JA, Talbot NJ (2010) An NADPH-dependent genetic switch regulates plant infection by the rice blast fungus. Proc Natl Acad Sci U S A 107(50):21902–21907. 10.1073/pnas.1006839107 21115813 10.1073/pnas.1006839107 PMC3003025

[CR37] Xie D, Tang C, Wang Y, Jin H, Wang Y (2023) The Transcription Factor CcRlm1 Regulates Cell Wall Maintenance and Poplar Defense Response by Directly Targeting *CcChs6* and *CcGna1* in *Cytospora chrysosperma*. Appl Environ Microbiol 89:e0066123. 10.1128/aem.00661-23 10.1128/aem.00661-23 PMC10304682 37289076

[CR38] Xu Z, Xiong D, Han Z, Tian C (2022) A putative effector CcSp84 of *Cytospora chrysosperma* localizes to the plant nucleus to trigger plant immunity. Int J Mol Sci 23(3):14. 10.3390/ijms23031614 10.3390/ijms23031614 PMC8835870 35163540

[CR39] Xu S, Li Q, Jin H, Li A, Wang Y (2025) Trehalose biosynthetic genes are involved in the development and pathogenesis in the poplar canker fungus *Cytospora chrysosperma*. Phytopathology 115(3):260–268. 10.1094/PHYTO-05-24-0160-R 39499502 10.1094/PHYTO-05-24-0160-R

[CR40] Yamada K, Mine A (2024) Sugar coordinates plant defense signaling. Sci Adv 10(4):eadk4131–eadk4131. 10.1126/sciadv.adk4131 38266087 10.1126/sciadv.adk4131 PMC10807812

[CR41] Yang SL, Yu P, Chung K (2016) The glutathione peroxidase-mediated reactive oxygen species resistance, fungicide sensitivity and cell wall construction in the citrus fungal pathogen *Alternaria alternata*. Environ Microbiol 18(3):923–935. 10.1111/1462-2920.13125 26567914 10.1111/1462-2920.13125

[CR42] Yin Z, Liu H, Li Z, Ke X, Dou D, Gao X, Song N, Dai Q, Wu Y, Xu JR, Kang Z, Huang L (2015) Genome sequence of Valsa canker pathogens uncovers a potential adaptation of colonization of woody bark. New Phytol 208(4):1202–1216. 10.1111/nph.13544 26137988 10.1111/nph.13544

[CR43] Yuan P, Qian W, Jiang L, Jia C, Ma X, Kang Z, Liu J (2021) A secreted catalase contributes to *Puccinia striiformis* resistance to host-derived oxidative stress. Stress Biol 1(1):22–22. 10.1007/s44154-021-00021-2 37676381 10.1007/s44154-021-00021-2 PMC10441885

[CR44] Zeng S, Yang R, Ming Z, Li A (2025) The two-component system RstB / RstA regulates virulence through the Fe^3+^-siderophore transport operon lpfetABC in the poplar canker bacterium *Lonsdalea populi*. Phytopathol Res 7(1):14. 10.1186/s42483-025-00377-7

[CR45] Zhang F, Chen S, Zhang C, Wang Z, Miao J, Dai T, Hao J, Liu X (2024) PsDMAP1/PsTIP60-regulated H4K16ac is required for ROS-dependent virulence adaptation of *Phytophthora sojae* on host plants. Proc Natl Acad Sci 122(1):e2413127122. 10.1073/pnas.2413127122 39793040 10.1073/pnas.2413127122 PMC11725902

[CR46] Zhang H, Shen X, Shen W, Zhang D, Huang X, Zhu K, Liu J, Li G (2024b) Glutathione peroxidase LtGPX3 contributes to oxidative stress tolerance, virulence, and plant defense suppression in the peach gummosis fungus *Lasiodiplodia theobromae*. Phytopathology Res 6(1):15. 10.1186/s42483-024-00224-1

[CR47] Zhang N, Lv F, Qiu F, Han D, Xu Y, Liang W (2023) Pathogenic fungi neutralize plant‐derived ROS via Srpk1 deacetylation. Embo J 42(9):EMBJ2022112634. 10.15252/embj.2022112634 10.15252/embj.2022112634 PMC10152141 36891678

[CR48] Zhao X, Wang Y, Yuan B, Zhao H, Wang Y, Tan Z, Wang Z, Wu H, Li G, Song W, Gupta R, Tsuda K, Ma Z, Gao X, Gu Q (2024) Temporally-coordinated bivalent histone modifications of *BCG1* enable fungal invasion and immune evasion. Nat Commun 15(1):231. 10.1038/s41467-023-44491-6 38182582 10.1038/s41467-023-44491-6 PMC10770383

[CR49] Zhu J, Schworer S, Berisa M, Kyung YJ, Ryu KW, Yi J, Jiang X, Cross JR, Thompson CB (2021) Mitochondrial NADP (H) generation is essential for proline biosynthesis. Science 372(6545):968. 10.1126/science.abd5491 33888598 10.1126/science.abd5491 PMC8241437

