# Supplementary figures and images for: CcAbl1 is required for virulence via regulating NADPH–glutathione-mediated redox balance in Cytospora chrysosperma
Source: Stress Biol. 2026 Apr 24;6(1):33. doi: 10.1007/s44154-026-00302-8 (PMC13109457; doi:10.1007/s44154-026-00302-8)

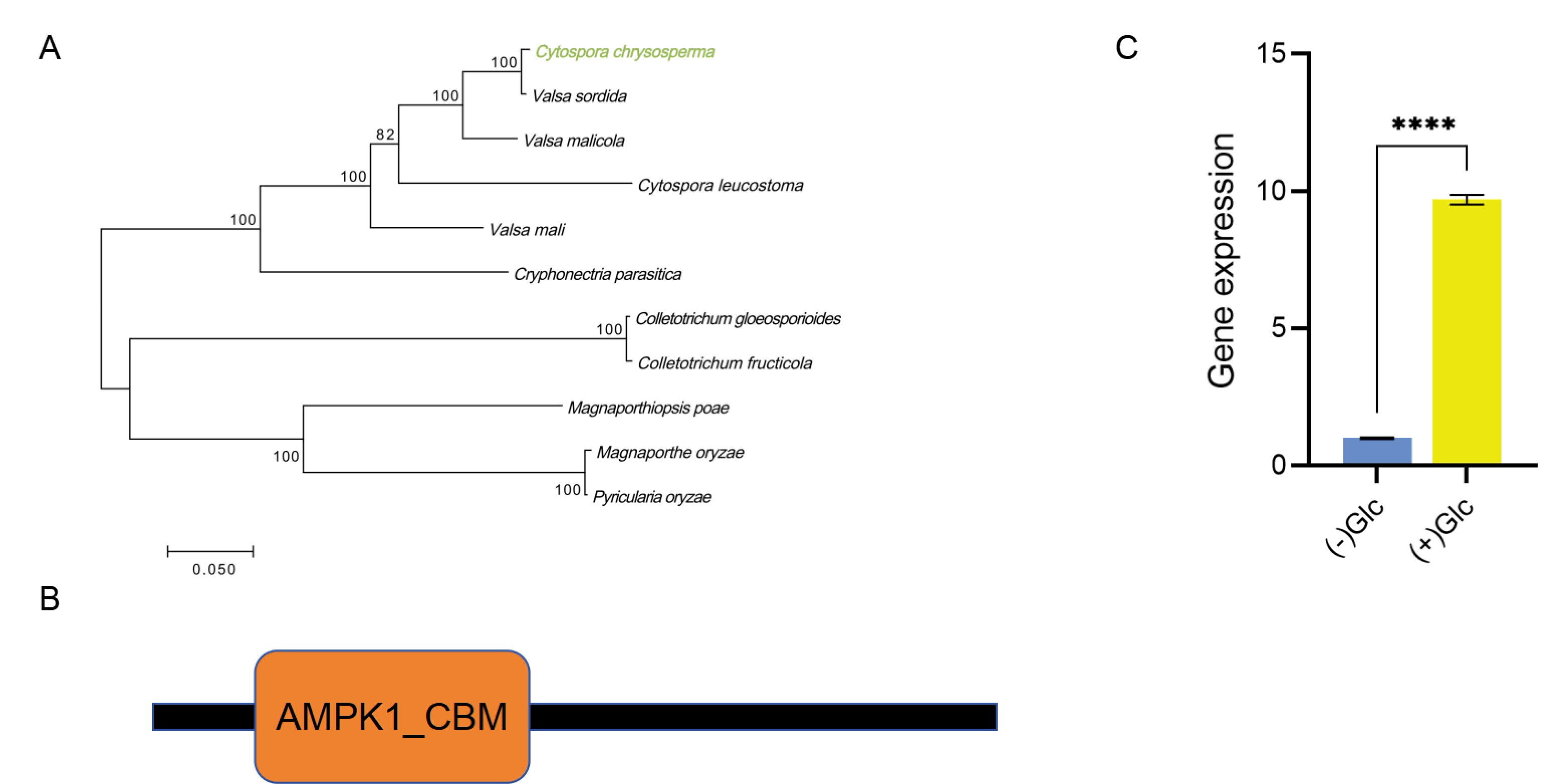

Supplement: Supplementary file 1 — Supplementary Material 1. [file 44154_2026_302_MOESM1_ESM.zip › folder/Fig.S1_ESM.png]

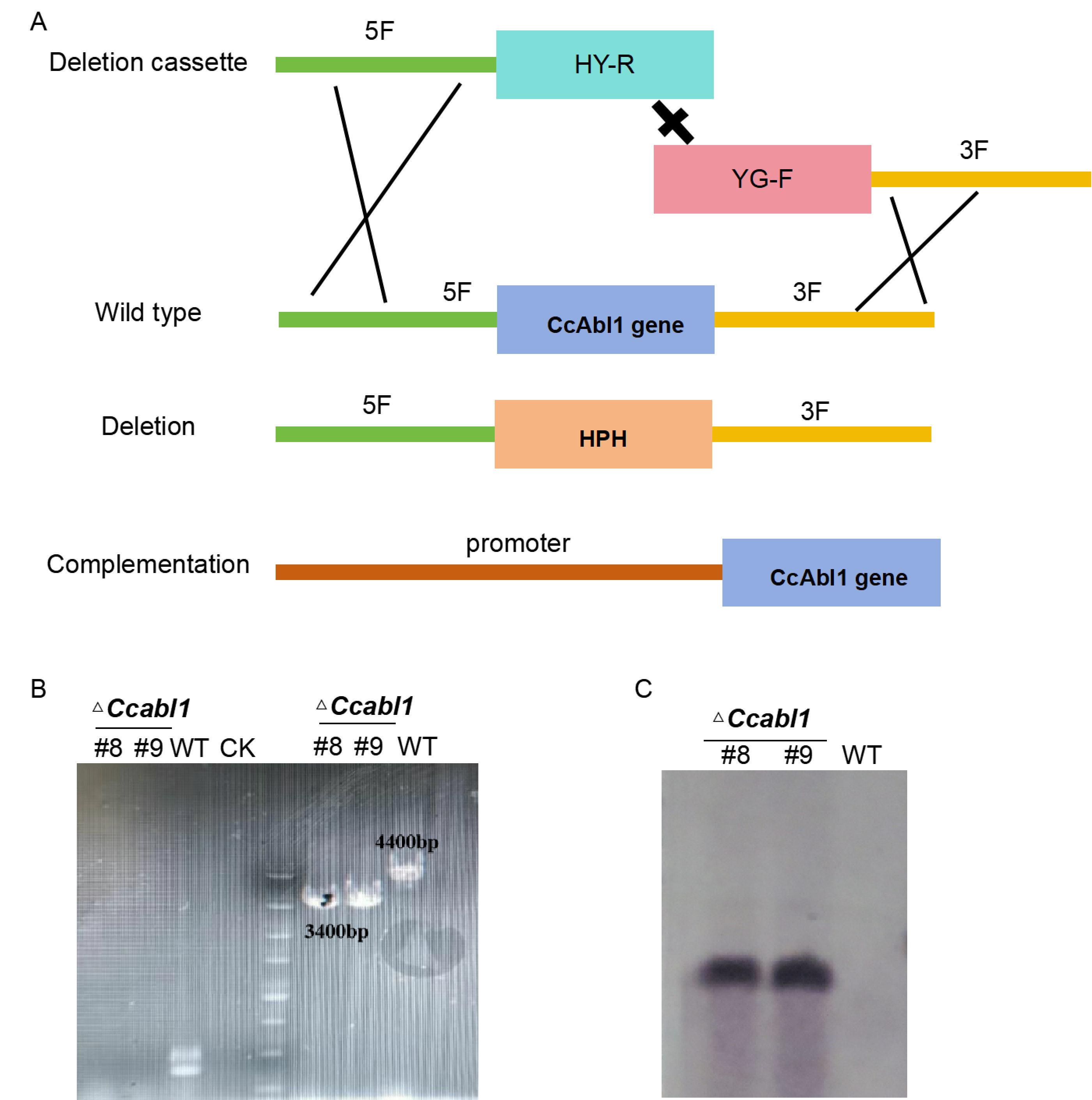

Supplement: Supplementary file 1 — Supplementary Material 1. [file 44154_2026_302_MOESM1_ESM.zip › folder/Fig.S2_ESM.png]

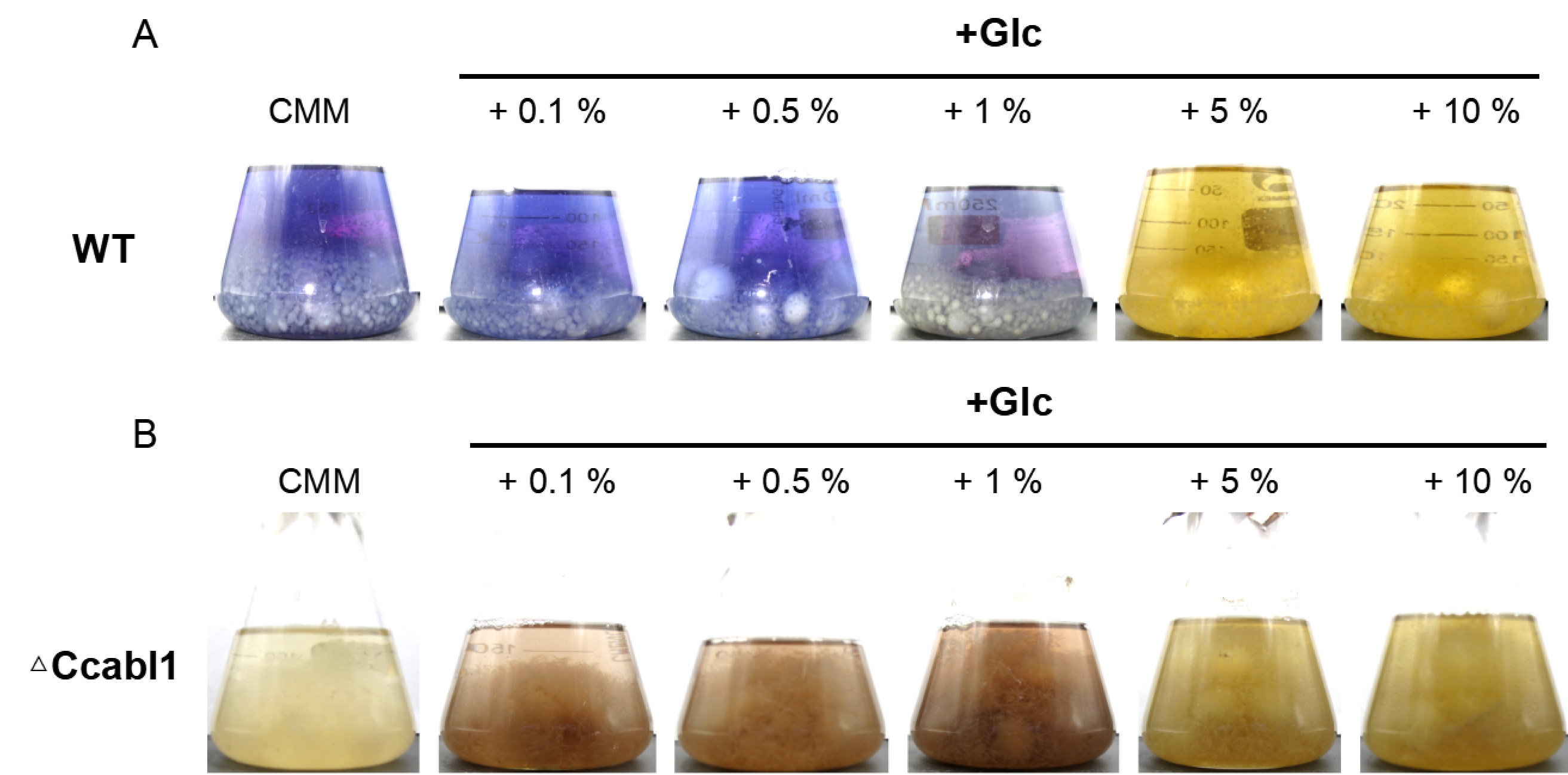

Supplement: Supplementary file 1 — Supplementary Material 1. [file 44154_2026_302_MOESM1_ESM.zip › folder/Fig.S3_ESM.png]

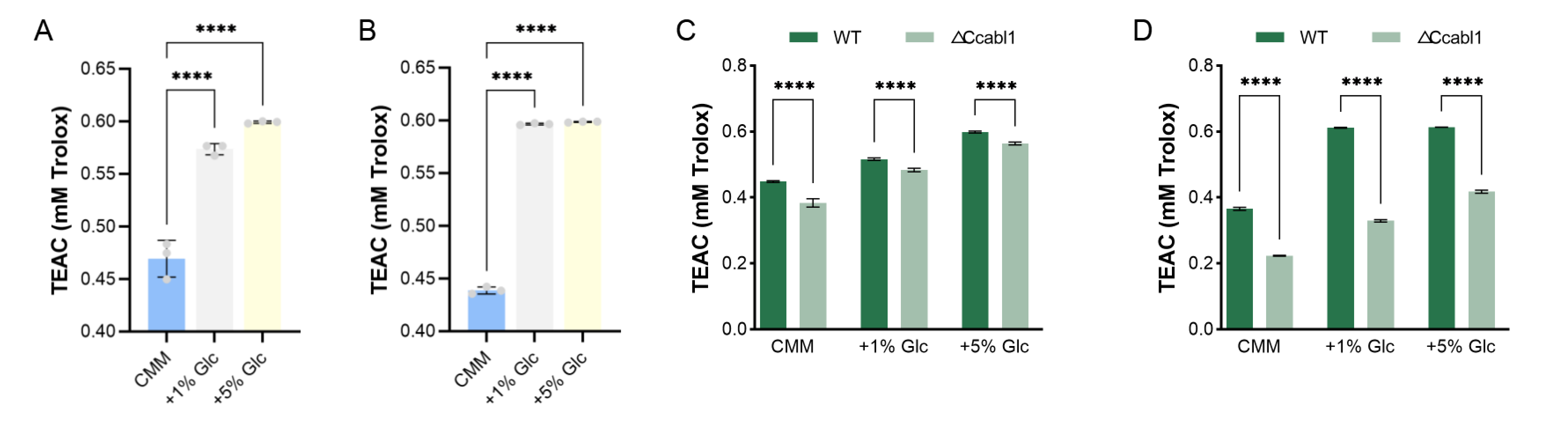

Supplement: Supplementary file 1 — Supplementary Material 1. [file 44154_2026_302_MOESM1_ESM.zip › folder/Fig.S4_ESM.png]

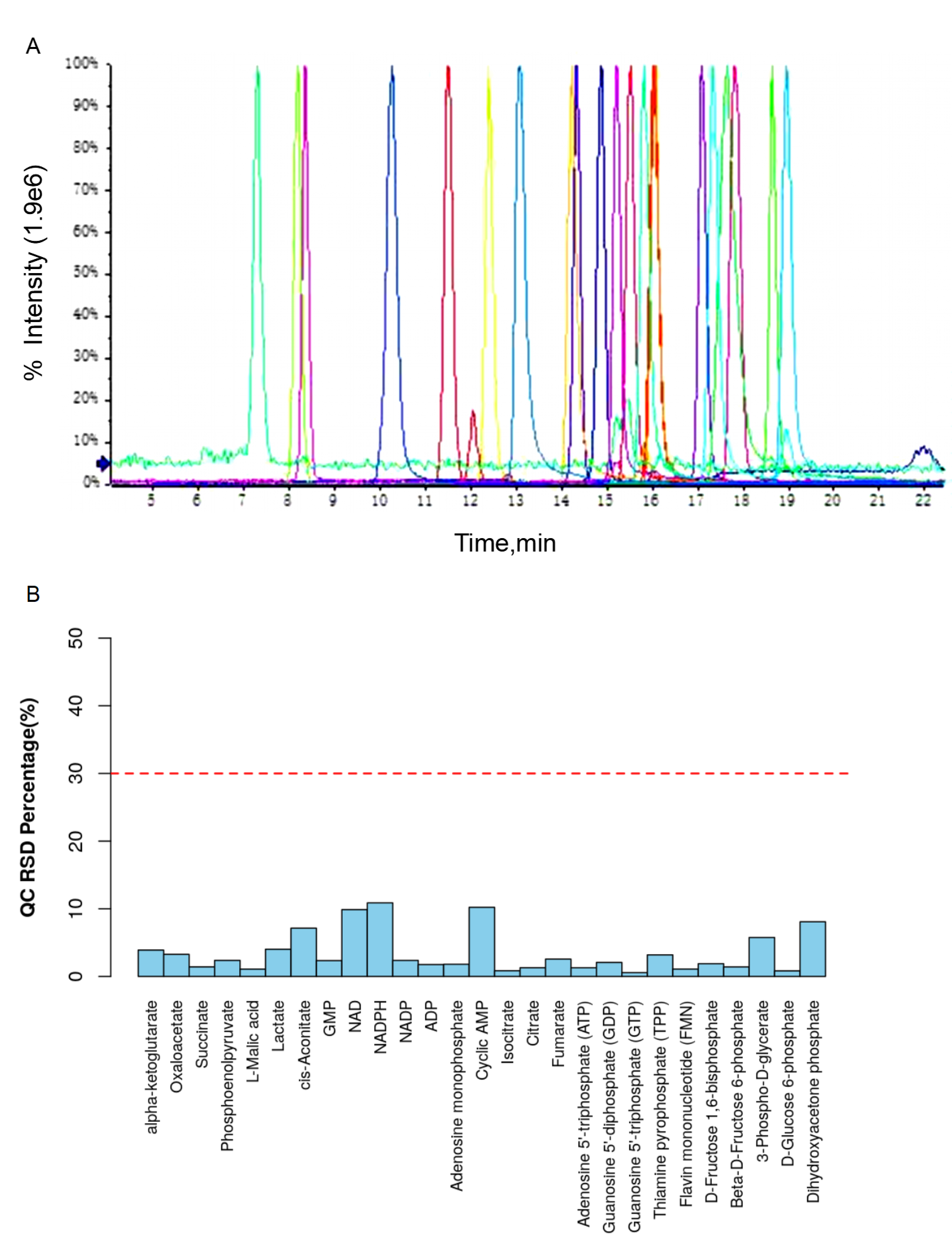

Supplement: Supplementary file 1 — Supplementary Material 1. [file 44154_2026_302_MOESM1_ESM.zip › folder/Fig.S5_ESM.png]

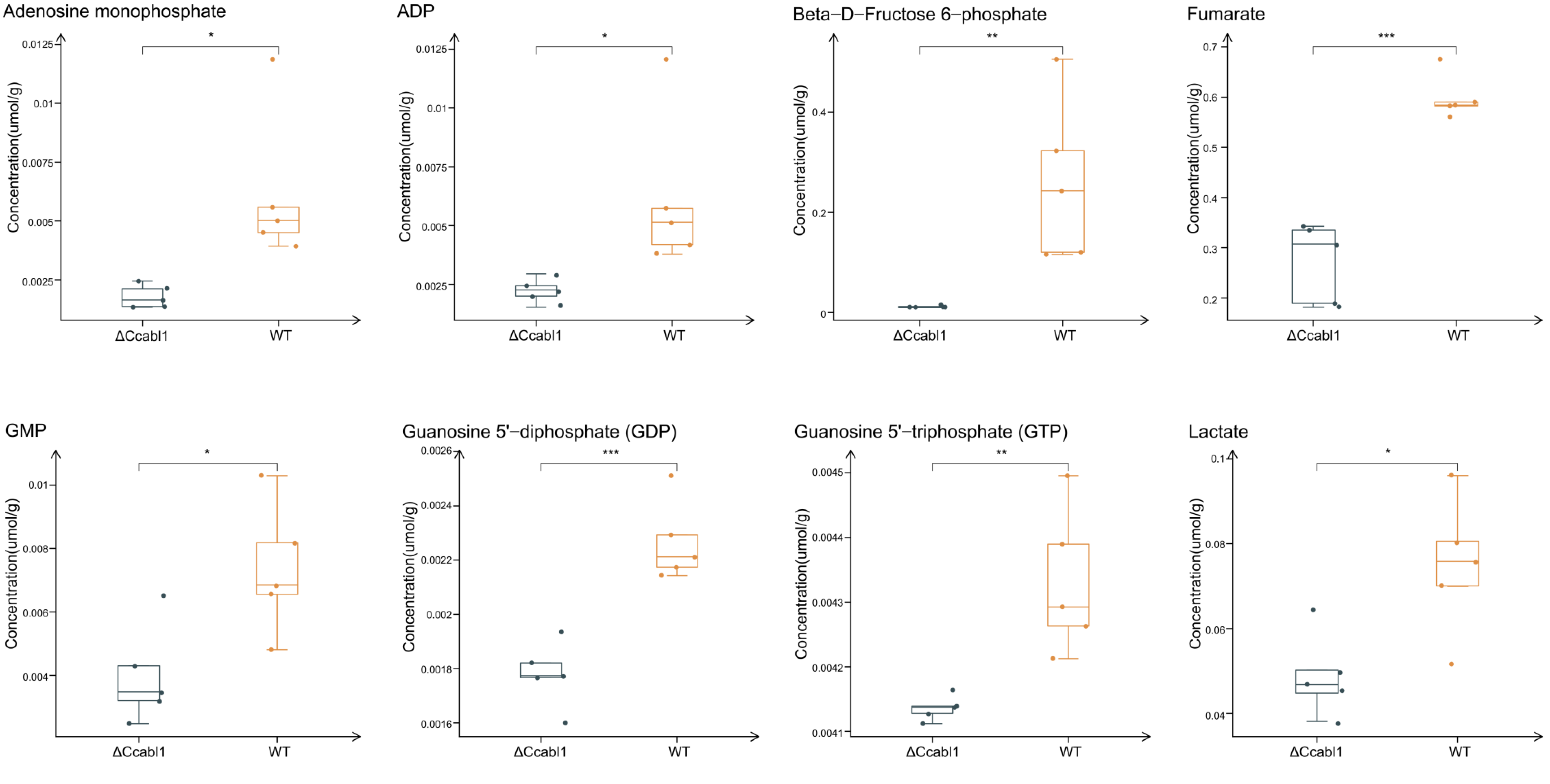

Supplement: Supplementary file 1 — Supplementary Material 1. [file 44154_2026_302_MOESM1_ESM.zip › folder/Fig.S6_ESM.png]

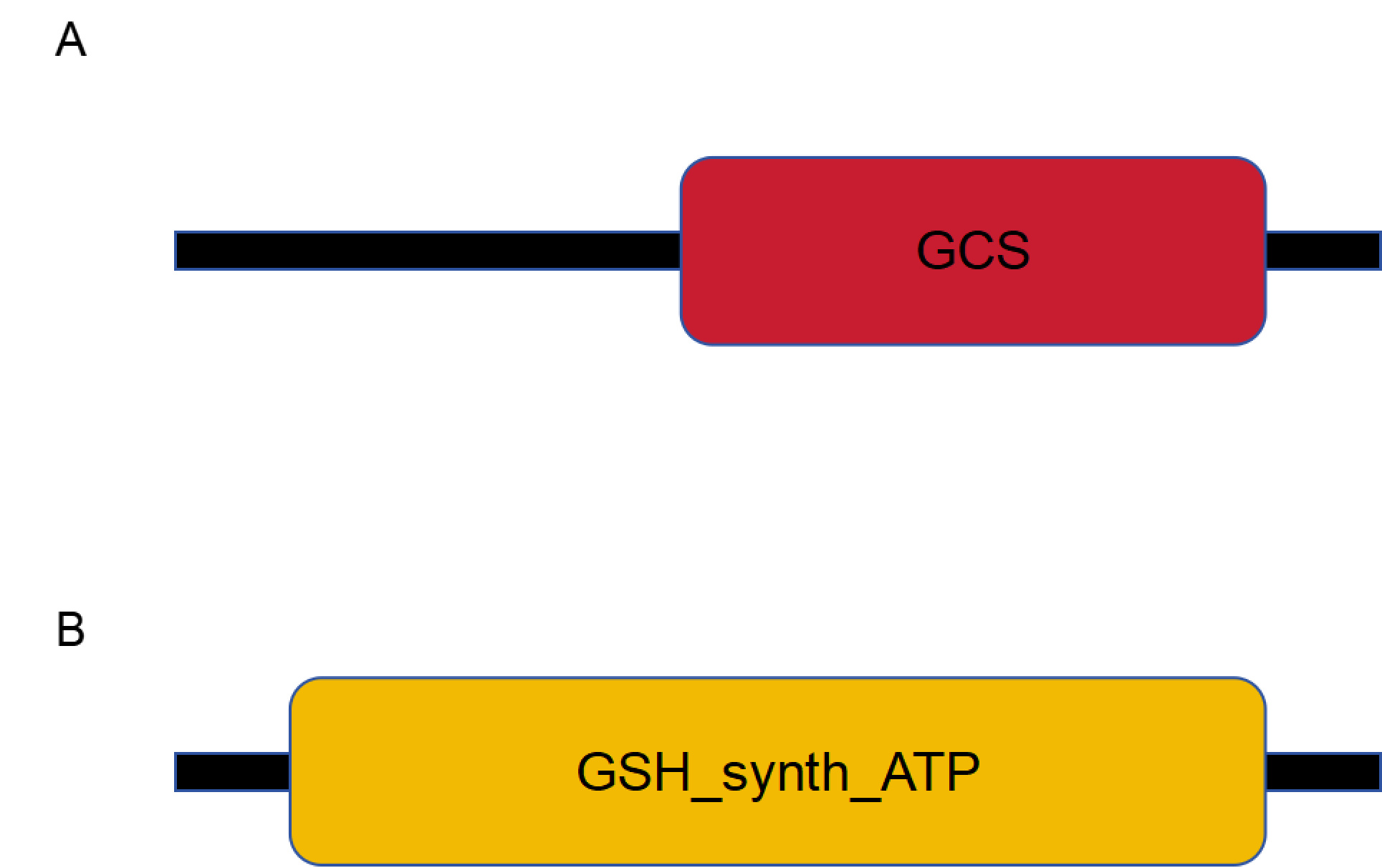

Supplement: Supplementary file 1 — Supplementary Material 1. [file 44154_2026_302_MOESM1_ESM.zip › folder/Fig.S7_ESM.png]

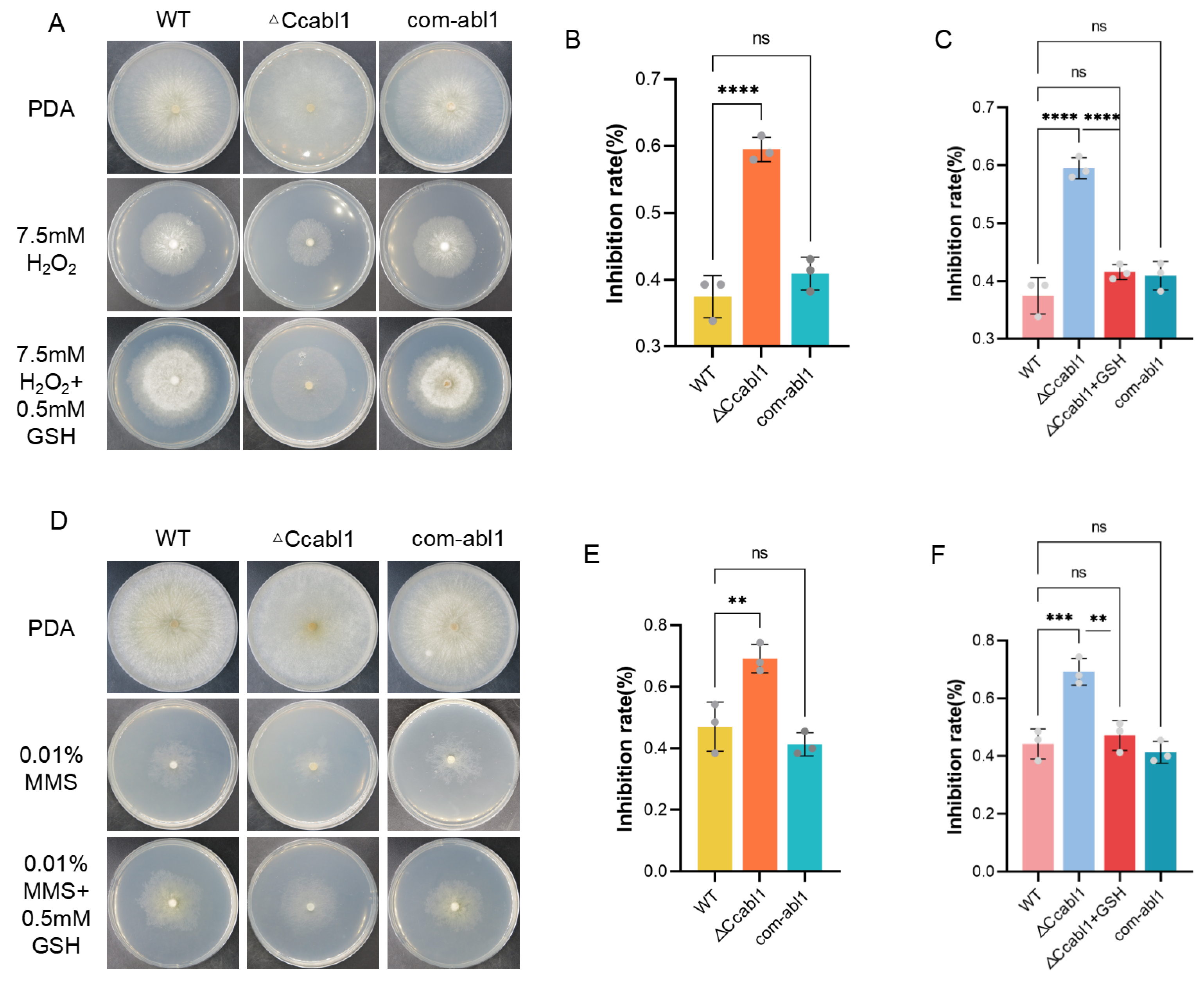

Supplement: Supplementary file 1 — Supplementary Material 1. [file 44154_2026_302_MOESM1_ESM.zip › folder/Fig.S8_ESM.png]
